# Different Metabolomic Responses to Carbon Starvation between Light and Dark Conditions in the Purple Photosynthetic Bacterium, *Rhodopseudomonas palustris*

**DOI:** 10.1264/jsme2.ME17143

**Published:** 2018-03-29

**Authors:** Nanako Kanno, Katsumi Matsuura, Shin Haruta

**Affiliations:** 1 Department of Biological Sciences, Tokyo Metropolitan University Minami-Osawa 1–1, Hachioji, Tokyo 192–0397 Japan

**Keywords:** starvation response, anoxygenic photosynthesis, cellular energy, non-growing phase, metabolome

## Abstract

Purple photosynthetic bacteria utilize light energy for growth. We previously demonstrated that light energy contributed to prolonging the survival of multiple purple bacteria under carbon-starved conditions. In order to clarify the effects of illumination on metabolic states under carbon-starved, non-growing conditions, we herein compared the metabolic profiles of starved cells in the light and dark using the purple bacterium, *Rhodopseudomonas palustris*. The metabolic profiles of starved cells in the light were markedly different from those in the dark. After starvation for 5 d in the light, cells showed increases in the amount of ATP and the NAD^+^/NADH ratio. Decreases in the amounts of most metabolites related to glycolysis and the TCA cycle in energy-rich starved cells suggest the active utilization of these metabolites for the modification of cellular components. Starvation in the dark induced the consumption of cellular compounds such as amino acids, indicating that the degradation of these cellular components produced ATP in order to maintain viability under energy-poor conditions. The present results suggest that intracellular energy levels alter survival strategies under carbon-starved conditions through metabolism.

Bacteria in natural environments frequently face nutrient limitations (12, 19, 26), which prevent the growth of most bacteria. Some bacteria survive by forming metabolically inactive endospores or cysts, while most bacterial species may maintain their viability without forming resting cells (7, 19, 25). Viable bacteria with low metabolic activities have been detected in freshwater, marine, soil, and subsurface environments (14, 15). In a recent review, Hoehler and Jørgensen (15) reported that the potential involvement of low-activity bacteria should not be neglected in contributing to biogeochemical cycles despite low metabolic activities. However, the metabolic states of bacteria under nutrient-starved and non-growing conditions have not yet been elucidated in detail.

In order to clarify cellular responses to nutrient and energy starvation, changes in gene expression and intracellular metabolic processes have been analyzed in many species of bacteria (2, 4, 22, 24, 27, 30, 31, 33). These studies showed marked differences in the transcriptional and metabolic profiles of bacteria between the exponentially growing and non-growing states. Chapman *et al.* (6) found that the adenylate energy charge (AEC) of nutrient-starved cells decreased with time, indicating that cellular energy is consumed under starved conditions. However, the effects of cellular energy levels on starvation responses have yet to be clarified.

Purple non-sulfur bacteria synthesize ATP using light via cyclic photophosphorylation, with no requirement for carbon or electron sources, and are widely distributed in natural environments (11, 21). We recently reported that illumination contributed to maintaining the viability of carbon-starved cells of purple non-sulfur photosynthetic bacteria: *Rhodopseudomonas palustris*, *Rhodobacter sphaeroides*, *Rhodospirillum rubrum*, and *Rubrivivax gelatinosus* (17). We also measured ATP levels in *R. palustris* under carbon-starved conditions, and found that cell viability was not decreased during starvation for 5 d in the light and dark. ATP levels were maintained in 7-d-starved cells in the light, but decreased by 6.4-fold in 5-d-starved cells in the dark (17). These findings suggested that *R. palustris* cells survived starvation conditions in the light and dark in different manners.

In the present study, we applied a metabolomic approach to clarify the effects of illumination on the metabolic states of carbon-starved cells of *R. palustris*. We focused on the glycolytic pathway, pentose phosphate pathway, TCA cycle, and amino acid and nucleic acid metabolism. We compared four cell types: 1) cells in the exponential growth phase (described hereafter as “growing cells”), 2) cells within 2 h of reaching the stationary phase due to carbon depletion (d0-starved cells), 3) starved cells maintained for 5 d under starvation conditions in the light (d5-light starved cells), and 4) starved cells maintained for 5 d under starvation conditions in the dark (d5-dark starved cells).

## Materials and Methods

### Bacterial strain and preparation of growing and starved cells

*R. palustris* strain CGA009 (=ATCC BAA-98) was used in the present study. This strain was grown in carbon-rich medium to obtain experimental cells. Carbon-rich medium (pH 7.0) contained (L^−1^) 5 g disodium succinate hexahydrate, 1 g (NH_4_)_2_SO_4_, 0.38 g KH_2_PO_4_, 0.39 g K_2_HPO_4_, 1 mL of a vitamin mixture (10), and 5 mL of a basal salt solution (10). A total of 120 mL of the medium was added to a 150-mL glass vial, which was then sealed with a butyl rubber stopper and aluminum seal. The gas phase of the vials was replaced with N_2_ gas. Cultures were cultivated at 30°C in a water bath under illumination (tungsten lamp with a 750-nm long pass filter; 600 J s^−1^ m^−2^, quantified by a pyranometer [LI-190SA; Meiwafosis, Tokyo, Japan]). Culture solutions were continuously agitated using magnetic stirrers. The bacterial growth of each culture was assessed by monitoring optical density at 660 nm. Bacterial cells at the exponential growth phase (approx. OD=0.2) were collected to obtain growing cells.

A “carbon-limited medium” was used to obtain starved cells. The amount of sodium succinate in carbon-rich medium was reduced to 0.5 g L^−1^ in order to prepare this medium. Growth in carbon-limited medium ceased at the exponential growth phase (approx. OD=0.3) due to carbon depletion. Cells collected within 2 h after the increase in OD stopped were defined as “d0-starved cells”. Some of these cells were additionally incubated at 30°C with agitation in the light, as described above, or in the dark for 5 d. These cells were designated as “d5-light starved cells” and “d5-dark starved cells”, respectively.

### NAD^+^/NADH ratio

Intracellular NAD^+^ and NADH were extracted and assayed using a fluorescent NAD/NADH detection kit (Cell Technology, Mountain View, CA, USA). Culture portions were collected with illumination or not. Two separate culture portions were required for the assessment of NAD^+^ and NADH. Two portions (1 mL each) were immediately cooled in ice-cold water and harvested by centrifugation. Pellets were resuspended in 200 μL of NADH or NAD extraction buffer. Two hundred microliters of NAD/NADH lysis buffer was then added to each tube followed by two rounds of a freeze-thaw cycle. Samples were heated at 60°C for 15 min and then cooled on ice. One hundred microliters of the reaction buffer and 200 μL of the opposite extraction buffer were added to tubes to neutralize the samples. Supernatants were obtained by spinning the lysates. NADH and NAD^+^ concentrations in supernatants were assessed after enzyme reactions according to the manufacturer’s instructions by measuring fluorescence intensity at an emission wavelength of 595 nm (550 nm excitation). Data were normalized to culture optical density at 660 nm.

### Analysis of metabolites by capillary electrophoresis-time-of-flight mass spectrometry (CE-TOFMS)

A metabolomic analysis using CE-TOFMS was performed by Human Metabolome Technologies (Tsuruoka, Japan). Experimental culture vials were cooled to 4°C for 5 min; culture vials for d5-dark starved cells were kept away from the light. Cells in 120 mL of each culture solution were collected by filtration using 0.4-μm pore-size isopore membrane filters (Millipore, Herts, UK) and washed twice with Milli-Q water. Cells were treated with 1,600 μL of methanol with ultrasonication for 30 s. The cell extract was then mixed with 1,100 μL of Milli-Q water containing an internal standard (H3304-1002; Human Metabolome Technologies) and left for 30 s. The extract was centrifuged at 2,300×*g* at 4°C for 5 min and then 1,600 μL of the supernatant was centrifugally filtered through a Millipore 5-kDa cut-off filter at 9,000×*g* at 4°C for 180 min. The filtrate was dried and re-suspended in 50 μL of Milli-Q water for the CE-TOFMS analysis.

CE-TOFMS was performed using an Agilent CE Capillary Electrophoresis System equipped with an Agilent 6210 Time of Flight mass spectrometer, Agilent 1100 isocratic HPLC pump, Agilent G1603A CE-MS adapter kit, and Agilent G1607A CE-ESI-MS sprayer kit (Agilent Technologies, Waldbronn, Germany). These systems were controlled by Agilent G2201AA ChemStation software version B.03.01 for CE (Agilent Technologies). The metabolites were analyzed using a fused silica capillary (50 μm i.d.×80 cm total length), with commercial electrophoresis buffer (Solution ID: H3301-1001 for the cation analysis and I3302-1023 for the anion analysis; Human Metabolome Technologies) as the electrolyte. The sample was injected at a pressure of 50 mbar for 10 s (approximately 10 nL) in the cation analysis and 25 s (approximately 25 nL) in the anion analysis. The spectrometer scanned from *m/z* 50 to 1,000. Peak areas were corrected using those of the internal standards. Each metabolite was identified and quantified based on peak information, including *m/z*, the migration time, and peak area, using MasterHands ver.2.13.0.8.h (developed at Keio University).

Peak areas were normalized to culture optical densities (5, 20). The concentrations of 60 metabolites related to essential metabolism were calculated using the concentration of each standard substrate.

### Statistical analysis

A principal components analysis (PCA) of metabolic profiles (32) was performed using the peak areas of the detected metabolites in MATLAB R2012b (MathWorks, Natick, MA, USA). Differences in the concentrations of metabolites among the cells prepared in the present study were evaluated for individual metabolites by Welch’s *t*-test. Three independent cultures for each condition were used for calculations. In calculations, the concentrations of undetected metabolites were regarded as zero.

Differences in the NAD^+^/NADH ratio between growing cells, d5-light starved cells, and d5-dark starved cells were analyzed using Tukey’s HSD post hoc tests after a one-way analysis of variance (ANOVA).

## Results

### Energy state and NAD^+^/NADH ratio of growing and starved cells in the light and dark

As shown in Table 1, we compared the cellular energy state for four different conditions of cells, *i.e.* growing cells, d0-starved cells, d5-light starved cells, and d5-dark starved cells (Table 1). The amounts of ATP, ADP and AMP were not significantly different between growing cells and d0-starved cells. AEC, which was calculated using the amounts of ATP, ADP, and AMP, was utilized to evaluate the cellular energy state. Growing cells and d0-starved cells had AEC values of 0.90 and 0.89, respectively. These values are similar to those reported in other bacteria in the exponential growth phase (6). The AEC value of d5-light starved cells was 0.93. However, the amount of ATP detected in d5-light starved cells increased by more than two-fold. Starvation for 5 d in the dark decreased the amount of ATP and increased those of ADP and AMP. As a result, d5-dark starved cells had a low AEC value of 0.27.

In order to calculate the NAD^+^/NADH ratio, cellular NADH and NAD^+^ were quantified in three independent cultures: growing cells, d5-light starved cells, and d5-dark starved cells (Table 1). The NAD^+^/NADH ratio was similar between d5-dark starved cells and growing cells at 13±2 and 17±4, respectively. d5-Light starved cells had a significantly higher value, *i.e.*, 40±4 (*P*<0.05).

### Metabolic profiles of growing and starved cells in the light and dark

**Fig. 1:**
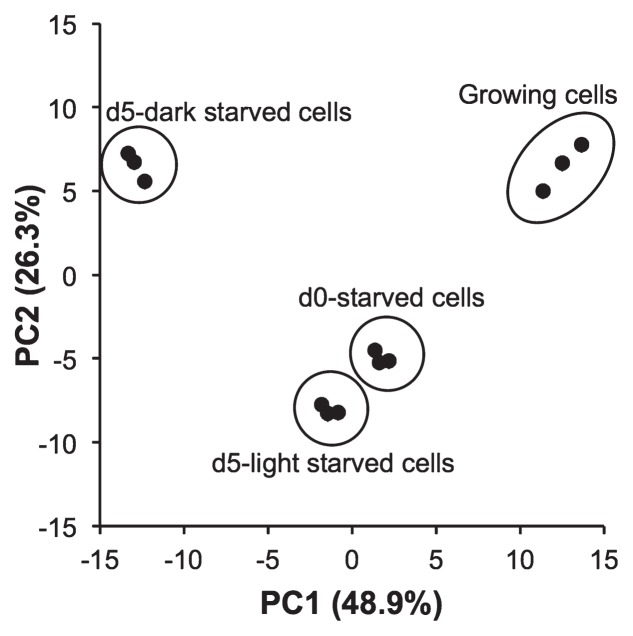
PCA scores plot for PC1 and PC2 for the comparison of metabolic profiles in growing cells, d0-starved cells, d5-light starved cells, and d5-dark starved cells. Each point represents a single culture. The peak areas of 183 metabolites in CE-TOFMS (Table S1) were used for calculations after normalization using the optical densities of the cultures.

Metabolomic analyses of the four types of cells identified a total of 183 different metabolites (Table S1). PCA was performed to infer similarities and differences among the metabolic profiles of each cell type (32). Fig. 1 shows the two-dimensional scores plot for principal component 1 (PC1) against PC2. PC1 and PC2 explained 48.9% and 26.3% of total variance, respectively. The scores plot clearly separated all four groups of samples. The PCA plot indicated that d0-starved cells differed largely from exponentially growing cells, but were similar to d5-light starved cells. As expected from the results obtained for energy level differences, d5-dark starved cells differed the most from the other cells. The scores plot was merged with the loadings plot of each metabolite (Fig. S1) in order to evaluate the relationship between the metabolic profile of each cell type and metabolites (32). The biplot of PCA shown in Fig. S1 indicates that growing cells differed from other cell types in their amounts of central metabolites and some amino acids. Nucleic acid metabolism markedly differed between d5-dark starved cells and others. Metabolites that markedly increased and decreased after starvation for 5 d in the dark included nucleoside monophosphates and triphosphates, respectively (Table S2). In contrast to starvation in the dark, marked differences in the amounts of metabolites were not observed between d5-light starved cells and d0-starved cells (Table S3).

### Differences in metabolite levels after starvation for 5 d in the light and dark

**Fig. 2:**
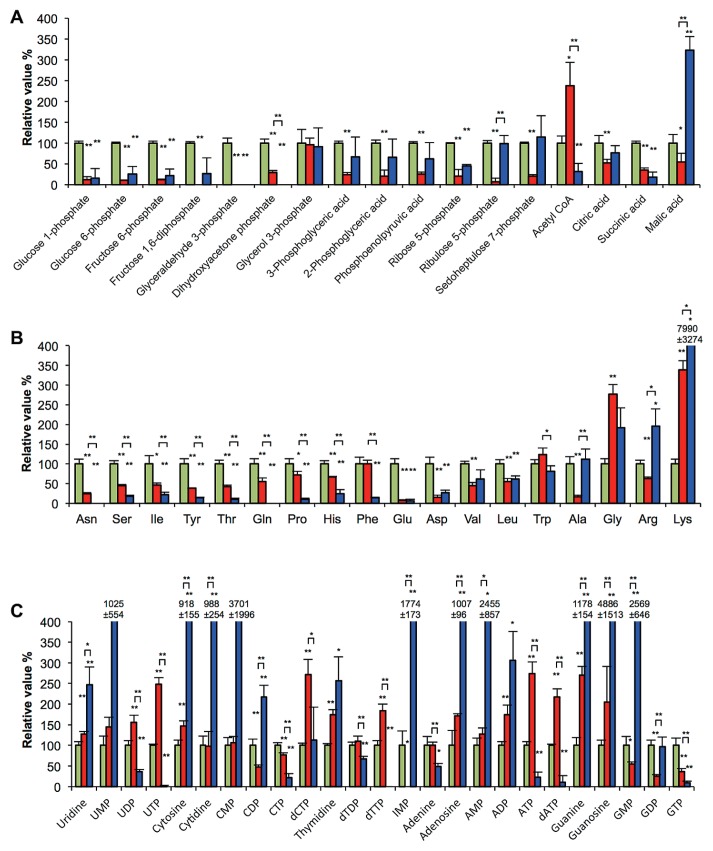
Differences in metabolite levels in cells starved for 5 d in the light and dark. Metabolite levels are shown as relative values, normalized to those of d0-starved cells as 100%. A, metabolites related to central metabolism such as the glycolytic pathway, pentose phosphate pathway, and TCA cycle; B, proteinogenic amino acids; C, metabolites related to nucleic acid metabolism. Green bar, d0-starved cells; red bar, d5-light starved cells; blue bar, d5-dark starved cells. Values are presented as the means of three independent cultures, and error bars represent standard deviations. *P* values are for a two-tailed *t*-test, 2-sample unequal variance (Welch’s *t*-test). *, 0.05<*P*<0.10; **, *P*<0.05. The concentrations of each metabolite are shown in Table S4. Pyruvic acid, isocitric acid, and 2-oxoglutaric acid were not detected in any sample tested in this study. Oxaloacetic acid was not included in this analysis.

In order to clarify differences in metabolism after 5 d of carbon starvation with or without illumination, we focused on the amounts of metabolites related to the following metabolic pathways: central carbon metabolism, protein biosynthesis/degradation, and nucleic acid metabolism. Sixty metabolites related to these metabolisms were quantified (see Table S4) and values relative to those of d0-starved cells are shown in Fig. 2.

Fig. 2A shows the results of the quantification of central carbon metabolites. The amounts of 6 metabolites in the first half of the glycolytic pathway (glucose 1-phosphate, glucose 6-phosphate, fructose 6-phosphate, fructose 1,6-diphosphate, glycerol 3-phosphate, and dihydroxyacetone phosphate) markedly decreased after starvation in the light as well as in the dark. The amounts of 3 metabolites in the latter half of the glycolytic pathway (3-phosphoglyceric acid, 2-phosphoglyceric acid, and phosphoenolpyruvic acid) significantly decreased after starvation in the light, but not in the dark. In the case of metabolites in the pentose phosphate pathway, 2 metabolites (ribulose 5-phosphate and sedoheptulose 7-phosphate) decreased in the light, but were maintained in the dark. The high-energy compound, acetyl-CoA markedly increased in the light. An increase in malic acid levels was observed in starved cells in the dark only; malic acid levels decreased in the light. No marked differences were noted in the amount of glycerol 3-phosphate detected in the light and dark. Pyruvic acid, isocitric acid, and 2-oxoglutaric acid were not detected in any sample.

Differences in proteinogenic amino acids among starvation conditions are shown in Fig. 2B. Eleven out of the 18 amino acids quantified decreased in the light and dark. Of these, the levels of 8 amino acids (Asn, Ser, Ile, Tyr, Thr, Gln, Pro, and His) were significantly lower in starved cells in the dark than in starved cells in the light. Starved cells in the dark also showed lower Phe, Trp, and Gly levels than those in starved cells in the light. On the other hand, Ala and Arg levels were higher in the dark than in the light. Lys markedly accumulated in cells starved in the light and dark, particularly in the dark; Lys levels in cells starved in the light and dark were 3- and 80-fold higher than those in d0-starved cells, respectively.

Fig. 2C shows difference in the metabolites related to nucleic acid metabolism. As with the increase in ATP shown in Table 1, other nucleoside triphosphates, such as UTP, dCTP, dTTP and dATP, also increased in the light. Starved cells in the dark were characterized by marked increases in nucleoside monophosphates including UMP, CMP, IMP, AMP, and GMP and their related nucleobases.

## Discussion

In the present study, we compared the metabolic states of an anoxygenic photosynthetic bacterium in the exponential growth phase and under varying conditions of starvation with and without illumination. A PCA analysis of metabolic profiles revealed marked differences between d5-light and d5-dark starved cells.

Differences in nucleic acid metabolites were observed between starved cells in the light and dark (Fig. S1). Starved cells in the light showed elevated levels of high-energy compounds, such as ATP, UTP, GTP, CTP and acetyl-CoA, and had a high AEC value (Table 1, Fig. 2). The levels of many metabolites related to the glycolytic pathway, pentose phosphate pathway, and TCA cycle were markedly decreased (Table 1, Figs. 2A and 2B), suggesting that these metabolites were utilized as sources for the modification of cellular components such as membrane fatty acids (23) supported by an energy supply from photosynthesis under carbon-starved conditions. The NAD^+^/NADH ratio was higher in starved cells in the light than in starved cells in the dark and in growing cells (Table 1). NADH appears to be actively utilized for biosynthesis in the light. Many species of amino acids were retained after starvation for 5 d in the light, but decreased in the dark. High levels of amino acids may contribute to protein synthesis in order to support adaptive responses in starved cells. Adaptive responses through new protein synthesis have been indicated in nitrogen-starved non-growing *R. palustris* cells in the light (1, 22). McKinlay *et al.* reported an increase in the transcription of a sigma factor (RPA4225) (22). Their subsequent proteomic study showed that this sigma factor induced the expression of proteins including catalase and the starvation-inducible DNA-binding protein Dps (1).

One of the metabolic characteristics of d5-dark starved cells was decreased levels of 12 amino acids (Asn, Ser, Ile, Tyr, Thr, Gln, Pro, His, Phe, Glu, Asp, and Leu) (Fig. 2B). In the dark under carbon-starved conditions, cells cannot acquire energy or organic compounds from extracellular sources, but maintain a certain energy level (Table 1, Fig. 2). Energy levels may have been maintained by the observed degradation of amino acids. The anaerobic utilization of amino acids has been shown to produce ATP through substrate level phosphorylation (29). In the present study, a decrease in threonine was observed in the dark (Fig. 2B), and may be related to ATP synthesis, as reported in *Escherichia coli* (13). In addition, a decrease in the amount of serine (Fig. 2B) may result in the production of acetyl-CoA through the reactions of serine dehydratase and pyruvate dehydrogenase. Acetyl-CoA has the ability to produce ATP via phosphate acetyltransferase and acetate kinase reactions (29), both of which have been detected in *R. palustris* (9). The lower NAD^+^/NADH ratio in starved cells in the dark also indicates the more active oxidation of cellular compounds from that in starved cells in the light (Table 1).

Lysine markedly accumulated after starvation for 5 d under light and dark conditions, and accumulated more in the dark (Fig. 2B). Lysine is a precursor of cadaverine, a polyamine associated with various biochemical processes, *e.g.*, the prevention of DNA damage (8, 18, 28). Thus, the accumulation of lysine in starved cells may increase stress resistance in non-growing cells.

Our analyses allowed us to compare metabolic profiles between growing and starved cells, similar to previous studies (3, 16). Fig. S2 shows metabolite levels in growing and d0-starved cells, as represented in Fig. 2. Many metabolites related to central metabolism including phosphoenolpyruvate (PEP) were reduced by carbon starvation. In contrast, PEP largely accumulated in the carbon-starved cells of *E. coli* and *Saccharomyces cerevisiae* grown on glucose (3). In these organisms, PEP may be primarily utilized for the phosphorylation of carbohydrates and carbon starvation suppressed their active consumption. Furthermore, carbon-starved *E. coli* accumulated various amino acids including Glu (3, 16), which was largely consumed in carbon-starved *R. palustris* (Fig. S2). Energy production through protein degradation appears to have been required for carbon-starved *E. coli*, but not for carbon-starved *R. palustris* in the light.

In natural habitats, bacteria are exposed to fluctuations in their micro-environment, *e.g.*, changes in available nutrients and energy sources. We found marked differences in the metabolic states of non-growing cells maintained in the light and dark. Our results suggest that the intracellular energy levels of cells alter survival strategies under nutrient-starvation conditions. When large amounts of energy are available, carbon compounds related to central metabolism are consumed for biosynthesis, *e.g.*, the modification of cellular membranes. On the other hand, even when cells are starved without an extracellular energy supply, a reduced level of cellular energy is maintained for several days, possibly through the degradation of cellular components such as amino acids. Although cells in this state were not able to maintain viability for longer than 5 d (17), the degradation of the photosynthetic apparatus was not observed (Fig. S3) and cells re-started ATP synthesis instantly when illumination was applied again (data not shown). The availability of energy was found to be important for bacterial survival in non-growing cells (17). The present results indicate that when cells have sufficient energy, they modify their components; however, when they lack an external energy supply and have insufficient energy, they appear to degrade cellular components in order to obtain the minimum requirement of energy for survival.

## Supplementary Material



## Figures and Tables

**Table 1:** Energy state and NAD^+^/NADH ratio of growing and starved cells

	Growing cells	d0-starved cells	d5-starved cells	

			Light	Dark
ATP (nM/OD660)	2,102±175	1,710±166	4,678±495	387±211
ADP (nM/OD660)	350±43	299±29	521±70	916±209
AMP (nM/OD660)	71±9	78±14	100±12	1,920±670

AEC*	0.90±0.003	0.89±0.01	0.93±0.01	0.27±0.11

NAD^+^ (nM/OD660)	474±62	—	236±10	128±2
NADH (nM/OD660)	28±3	—	6.0±0.8	9.9±1.5

NAD^+^/NADH	17±4	—	40±4	13±2

* AEC, adenylate energy charge

The amounts of ATP, ADP, and AMP were assessed by a metabolomic analysis. NAD^+^ and NADH extracted from starved and growing cells were quantified. These values were normalized by the optical density value of the cultures. AEC values were calculated from the formula AEC=([ATP]+0.5[ADP])/([ATP]+[ADP]+[AMP]). Data are presented as means with standard deviations among three independent cultures.
