# Supplementary material for: Different Metabolomic Responses to Carbon Starvation between Light and Dark Conditions in the Purple Photosynthetic Bacterium, Rhodopseudomonas palustris
Source: Microbes Environ. 2018 Mar 29;33(1):83–8. doi: 10.1264/jsme2.ME17143 (PMC5877347; doi:10.1264/jsme2.ME17143)
Supplement: Supplementary file 1 [file 33_83_s1.pdf]

Supplementary material

Different Metabolomic Responses to Carbon Starvation between Light and Dark Conditions in the Purple Photosynthetic Bacterium, *Rhodopseudomonas palustris*

NANAKO KANNO[1], KATSUMI MATSUURA[1], and SHIN HARUTA[1]

[1]Department of Biological Sciences, Tokyo Metropolitan University, Minami-Osawa 1-1, Hachioji, Tokyo 192-0397, Japan

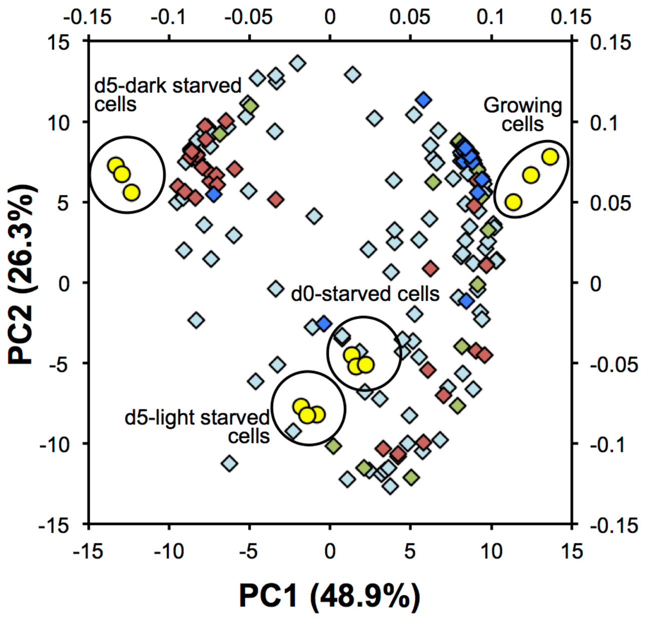

Fig. S1 Biplot of PCA for PC1 and PC2 for the comparison of metabolites in the growing cells, the d0-starved cells, the d5-light starved cells and the d5-dark starved cells. Circle-shaped dots in yellow color show scores of each cell and square-shaped dots show loadings of each metabolite. The loadings were calculated from the average value of 3 individual cultures. Blue, metabolites related to central metabolism; green, proteinogenic amino acids; red, metabolites related to nucleic acids metabolism; light blue, other metabolites.

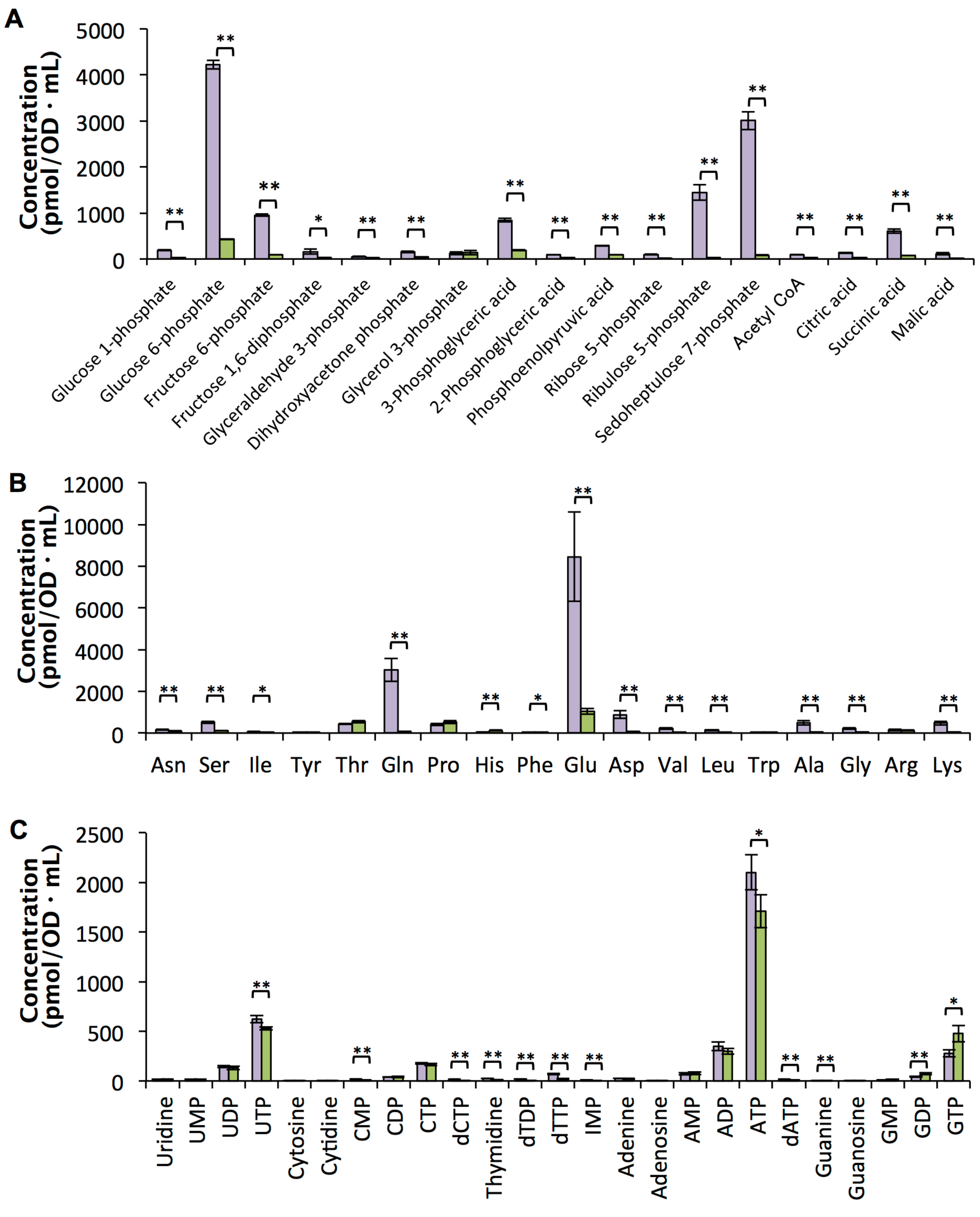

Fig. S2 Differences of metabolite concentrations between growing cells and starved cells. A, metabolites related to central metabolism such as glycolytic pathway, pentose phosphate pathway and TCA cycle; B, proteinogenic amino acids; C, metabolites related to nucleic acid metabolism. Purple bar, the growing cells; Green bar, the d0-starved cells. Values are presented as the means of three independent cultures,

and error bars represent standard deviations. *P* values are for two-tailed *t*-test, 2-sample unequal variance (Welch's t-test). *, $0.05 < p < 0.10$; **, $p < 0.05$. Concentrations of each metabolite are shown in Table S4.

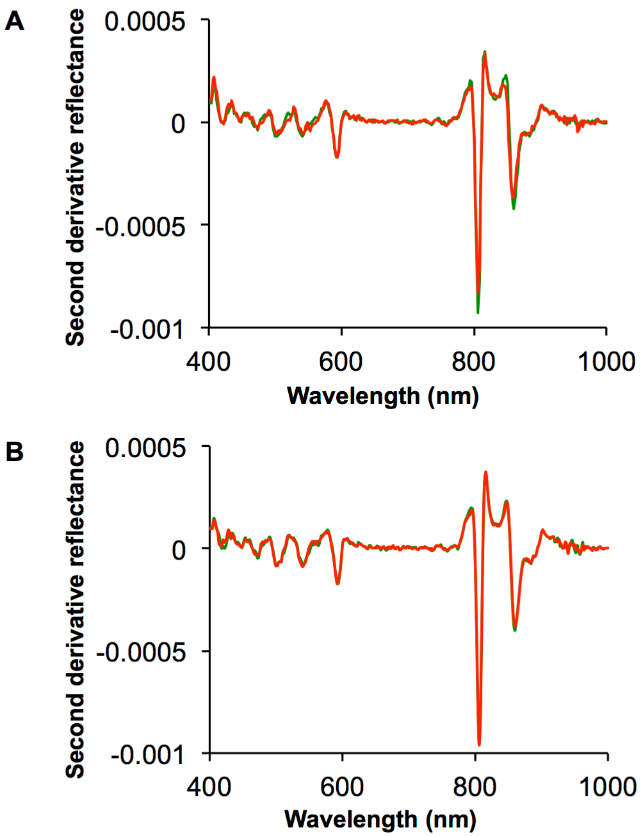

Fig. S3 Second derivative spectra of the cultures. A, d5-light starved cells (red line) and d0-starved cells (green line); B, d5-dark starved cells (red line) and d0-starved cells (green line). The second derivatives of the absorption spectrum with respect to wavelength were obtained by the Convolution (Savitzky-Golay) method using UVProve 2.42 software. The starved cells of *R. palustris* showed typical two absorption peaks of bacteriochlorophyll at 806 and 860 nm, which correspond to the absorption peaks of bacteriochlorophylls bound to the light-harvesting photopigment complexes.

Table S1 List of metabolites detected with CE-TOFMS and their peak area. These data were used for Fig. 1, Fig. S1 and Table S2, 3.

						Peak area per optical density of culture											
						Growing cells			d0-starved cells			d5-light starved cells			d5-dark starved cells		
Category*	Compound name	KEGG ID	HMDB ID	*m/z*	MT	1	2	3	1	2	3	1	2	3	1	2	3
Central	Succinic acid	C00042	HMDB00254	117.019	21.29	3.4E-02	2.9E-02	3.4E-02	4.3E-03	4.0E-03	4.4E-03	1.6E-03	1.7E-03	1.3E-03	1.2E-03	1.1E-03	N.D.
Central	Malic acid	C00149,C00497,C00711	HMDB00156,HMDB00744	133.014	21.75	6.5E-03	7.7E-03	1.1E-02	7.6E-04	1.1E-03	1.3E-03	8.9E-04	4.1E-04	4.5E-04	2.9E-03	3.7E-03	3.6E-03
Central	Phosphoenolpyruvic acid	C00074	HMDB00263	166.974	21.46	1.1E-02	1.0E-02	1.1E-02	3.2E-03	3.4E-03	3.5E-03	1.1E-03	7.2E-04	8.1E-04	1.1E-03	1.2E-03	3.9E-03
Central	Dihydroxyacetone phosphate	C00111	HMDB01473	168.990	12.63	3.5E-03	4.2E-03	5.0E-03	1.1E-03	9.6E-04	1.2E-03	3.3E-04	3.8E-04	2.4E-04	N.D.	N.D.	N.D.
Central	Glyceraldehyde 3-phosphate	C00118,C00661	HMDB01112	168.991	11.56	6.0E-04	5.6E-04	4.3E-04	2.3E-04	2.5E-04	1.9E-04	N.D.	N.D.	N.D.	N.D.	N.D.	N.D.
Central	Glycerol 3-phosphate	C00093	HMDB00126	171.005	12.06	4.8E-03	4.5E-03	2.7E-03	5.9E-03	5.1E-03	2.5E-03	3.4E-03	4.7E-03	4.9E-03	2.2E-03	3.2E-03	6.9E-03
Central	3-Phosphoglyceric acid	C00197	HMDB00807	184.985	19.91	2.5E-02	2.5E-02	2.8E-02	5.6E-03	5.9E-03	6.2E-03	1.6E-03	1.1E-03	1.7E-03	2.0E-03	2.0E-03	7.9E-03
Central	2-Phosphoglyceric acid	C00631	HMDB03391	184.985	19.44	2.8E-03	3.0E-03	3.3E-03	9.0E-04	8.3E-04	7.5E-04	N.D.	2.2E-04	2.8E-04	2.8E-04	2.9E-04	1.1E-03
Central	Citric acid	C00158	HMDB00094	191.018	26.97	1.2E-02	1.1E-02	9.6E-03	2.6E-03	1.7E-03	2.1E-03	1.4E-03	9.9E-04	1.0E-03	1.9E-03	1.9E-03	1.1E-03
Central	Ribose 5-phosphate	C00117	HMDB01548	229.010	10.46	4.4E-03	4.3E-03	4.5E-03	8.4E-04	8.1E-04	8.1E-04	N.D.	2.8E-04	2.4E-04	3.6E-04	3.3E-04	4.1E-04
Central	Ribulose 5-phosphate	C00199,C01101	HMDB00618	229.011	10.90	4.8E-02	5.5E-02	6.5E-02	1.0E-03	1.2E-03	1.2E-03	2.2E-04	N.D.	N.D.	8.7E-04	1.0E-03	1.4E-03
Central	Fructose 6-phosphate	C05345,C00085	HMDB00124	259.021	9.86	4.3E-02	4.3E-02	4.0E-02	4.2E-03	4.1E-03	3.7E-03	5.1E-04	5.0E-04	4.2E-04	4.3E-04	4.0E-04	1.8E-03
Central	Glucose 6-phosphate	C00668,C01172,C00092	HMDB01401	259.021	9.75	1.7E-01	1.8E-01	1.8E-01	1.8E-02	1.8E-02	1.8E-02	1.8E-03	1.9E-03	2.0E-03	2.4E-03	1.9E-03	9.4E-03
Central	Glucose 1-phosphate	C00103	HMDB01586	259.021	10.04	7.8E-03	8.5E-03	7.9E-03	1.1E-03	9.6E-04	9.6E-04	N.D.	1.6E-04	1.9E-04	N.D.	N.D.	4.7E-04
Central	Sedoheptulose 7-phosphate	C05382	HMDB01068	289.032	9.51	1.5E-01	1.4E-01	1.3E-01	4.1E-03	3.8E-03	3.9E-03	9.2E-04	6.2E-04	8.7E-04	2.9E-03	3.2E-03	7.4E-03
Central	Ribulose 1,5-diphosphate	C01182	No ID	308.978	15.75	N.D.	N.D.	N.D.	2.5E-04	1.5E-04	1.9E-04	N.D.	N.D.	N.D.	4.1E-04	6.3E-04	1.5E-03
Central	Fructose 1,6-diphosphate	C00354	HMDB01058	338.987	14.62	4.6E-03	7.3E-03	1.1E-02	1.8E-03	1.7E-03	1.7E-03	N.D.	N.D.	N.D.	N.D.	N.D.	1.4E-03
Central	Acetyl CoA_divalent	C00024	HMDB01206	403.554	10.15	6.4E-03	6.2E-03	6.1E-03	2.4E-03	1.6E-03	2.1E-03	6.5E-03	4.1E-03	4.1E-03	1.0E-03	8.5E-04	1.2E-04
Amino acid	Gly	C00037	HMDB00123	76.039	8.39	1.6E-02	2.1E-02	2.6E-02	3.8E-03	5.3E-03	4.9E-03	1.2E-02	1.3E-02	1.4E-02	1.1E-02	9.8E-03	5.8E-03
Amino acid	Ala	C00041,C00133,C01401	HMDB00161,HMDB01310	90.055	9.13	6.1E-02	9.5E-02	1.1E-01	9.1E-03	6.4E-03	6.2E-03	1.6E-03	1.0E-03	1.0E-03	9.8E-03	9.0E-03	5.6E-03
Amino acid	Ser	C00065,C00716,C00740	HMDB00187,HMDB03406	106.050	10.11	5.8E-02	5.6E-02	6.8E-02	1.1E-02	1.4E-02	1.3E-02	6.2E-03	5.3E-03	5.6E-03	2.5E-03	2.5E-03	2.1E-03
Amino acid	Pro	C00148,C00763,C16435	HMDB00162,HMDB03411	116.070	10.96	1.1E-01	1.2E-01	1.4E-01	1.4E-01	1.8E-01	1.3E-01	1.0E-01	9.8E-02	1.3E-01	2.0E-02	1.6E-02	1.0E-02
Amino acid	Val	C00183,C06417,C16436	HMDB00883	118.086	10.14	5.3E-02	7.9E-02	7.9E-02	6.5E-03	7.5E-03	6.4E-03	3.7E-03	3.2E-03	2.3E-03	3.0E-03	3.2E-03	6.4E-03
Amino acid	Thr	C00188,C00820	HMDB00167	120.065	10.66	7.9E-02	7.5E-02	8.5E-02	9.2E-02	1.1E-01	9.4E-02	4.8E-02	3.9E-02	4.2E-02	1.3E-02	1.1E-02	7.1E-03
Amino acid	Ile	C00407,C06418,C16434	HMDB00172	132.102	10.34	1.6E-02	2.7E-02	3.1E-02	7.4E-03	1.2E-02	1.2E-02	5.5E-03	4.8E-03	4.4E-03	2.0E-03	1.8E-03	3.2E-03
Amino acid	Leu	C00123,C01570,C16439	HMDB00687	132.102	10.45	5.5E-02	8.4E-02	7.6E-02	8.9E-03	1.2E-02	1.0E-02	6.8E-03	5.3E-03	4.9E-03	5.4E-03	6.2E-03	7.3E-03
Amino acid	Asn	C00152,C01905,C16438	HMDB00168	133.061	10.63	1.8E-02	2.3E-02	2.3E-02	1.2E-02	1.5E-02	1.2E-02	3.6E-03	3.3E-03	2.6E-03	N.D.	N.D.	N.D.
Amino acid	Asp	C00049,C00402,C16433	HMDB00191,HMDB06483	134.045	11.74	1.2E-01	1.1E-01	1.7E-01	6.9E-03	9.9E-03	1.0E-02	2.1E-03	1.2E-03	1.0E-03	3.1E-03	2.7E-03	1.7E-03
Amino acid	Gln	C00064,C00303,C00819	HMDB00641,HMDB03423	147.076	10.91	4.1E-01	4.6E-01	6.2E-01	1.0E-02	1.4E-02	1.2E-02	7.9E-03	6.5E-03	5.5E-03	N.D.	N.D.	N.D.
Amino acid	Lys	C00047,C00739,C16440	HMDB00182,HMDB03405	147.112	6.91	5.7E-02	8.2E-02	9.3E-02	4.4E-03	5.0E-03	5.9E-03	1.7E-02	1.6E-02	1.9E-02	5.7E-01	4.6E-01	1.8E-01
Amino acid	Glu	C00025,C00217,C00302	HMDB00148,HMDB03339	148.060	11.10	9.2E-01	1.4E+00	1.8E+00	1.4E-01	1.7E-01	2.0E-01	1.5E-02	1.3E-02	1.3E-02	1.6E-02	1.4E-02	3.0E-03
Amino acid	His	C00135,C00768,C06419	HMDB00177	156.077	7.34	9.8E-03	9.3E-03	9.8E-03	2.4E-02	2.9E-02	2.7E-02	1.8E-02	1.8E-02	1.8E-02	9.3E-03	7.5E-03	2.9E-03
Amino acid	Phe	C00079,C02057,C02265	HMDB00159	166.086	11.25	5.0E-03	4.8E-03	4.9E-03	6.1E-03	8.3E-03	9.3E-03	8.9E-03	7.9E-03	7.1E-03	9.8E-04	1.1E-03	1.3E-03
Amino acid	Arg	C00062,C00792	HMDB00517,HMDB03416	175.119	7.15	2.8E-02	3.3E-02	4.6E-02	3.0E-02	3.3E-02	2.6E-02	1.9E-02	1.7E-02	2.0E-02	7.0E-02	6.4E-02	4.0E-02
Amino acid	Tyr	C00082,C01536,C06420	HMDB00158	182.081	11.53	4.1E-03	4.3E-03	5.7E-03	3.6E-03	4.8E-03	4.9E-03	1.7E-03	1.7E-03	1.7E-03	7.3E-04	6.5E-04	6.0E-04
Amino acid	Trp	C00078,C00525,C00806	HMDB00929	205.098	11.17	3.4E-04	4.5E-04	4.7E-04	4.3E-04	5.5E-04	5.2E-04	6.1E-04	5.2E-04	7.3E-04	5.0E-04	3.8E-04	3.4E-04
Nucleic acid	dCMP	C00239	HMDB01202	306.048	9.63	N.D.	N.D.	N.D.	N.D.	N.D.	N.D.	1.3E-04	9.0E-05	N.D.	6.5E-04	1.2E-03	2.4E-03
Nucleic acid	dTMP	C00364	HMDB01227	321.048	9.43	N.D.	N.D.	N.D.	N.D.	N.D.	N.D.	N.D.	N.D.	N.D.	2.6E-04	3.4E-04	6.9E-04
Nucleic acid	CMP	C00055	HMDB00095	322.043	9.48	7.1E-04	6.9E-04	7.2E-04	3.2E-04	3.9E-04	5.1E-04	5.1E-04	4.3E-04	3.7E-04	6.9E-03	1.2E-02	2.6E-02
Nucleic acid	UMP	C00105	HMDB00288	323.027	9.68	5.0E-04	7.3E-04	9.0E-04	5.8E-04	5.0E-04	8.3E-04	9.6E-04	7.2E-04	1.1E-03	3.1E-03	5.1E-03	1.1E-02
Nucleic acid	AMP	C00020	HMDB00045	346.054	9.15	3.8E-03	3.9E-03	5.0E-03	4.5E-03	3.7E-03	5.7E-03	5.9E-03	5.1E-03	6.7E-03	6.8E-02	1.1E-01	1.6E-01
Nucleic acid	IMP	C00130	HMDB00175	347.037	9.45	3.5E-04	2.8E-04	3.0E-04	8.8E-05	1.7E-04	8.1E-05	N.D.	N.D.	N.D.	2.1E-03	2.1E-03	1.7E-03
Nucleic acid	GMP	C00144	HMDB01397	362.049	9.03	3.0E-04	3.0E-04	4.4E-04	4.6E-04	4.0E-04	6.5E-04	2.7E-04	2.5E-04	3.1E-04	9.0E-03	1.3E-02	1.7E-02
Nucleic acid	dTDP	C00363	HMDB01274	401.015	11.22	1.1E-03	1.0E-03	1.1E-03	3.5E-04	2.9E-04	3.0E-04	3.1E-04	3.2E-04	4.0E-04	2.2E-04	2.3E-04	1.8E-04
Nucleic acid	CDP	C00112	HMDB01546	402.010	11.37	1.8E-03	1.6E-03	1.7E-03	1.3E-03	1.8E-03	1.9E-03	7.6E-04	7.1E-04	9.2E-04	3.2E-03	4.3E-03	3.4E-03
Nucleic acid	UDP	C00015	HMDB00295	402.993	11.58	7.7E-03	7.5E-03	6.7E-03	6.0E-03	6.1E-03	7.7E-03	1.0E-02	9.0E-03	1.2E-02	2.2E-03	2.8E-03	2.3E-03

Nucleic acid	ADP	C00008	HMDB01341	426.021	10.77	2.4E-02	2.1E-02	1.8E-02	1.8E-02	1.6E-02	2.0E-02	3.3E-02	2.5E-02	3.5E-02	5.3E-02	7.1E-02	4.0E-02
Nucleic acid	GDP	C00035	HMDB01201	442.016	10.53	2.5E-03	2.2E-03	2.0E-03	4.3E-03	3.2E-03	4.1E-03	1.1E-03	8.1E-04	9.7E-04	3.5E-03	4.9E-03	2.7E-03
Nucleic acid	dCTP	C00458	HMDB00998	465.982	12.37	5.0E-04	6.3E-04	7.5E-04	2.0E-04	1.9E-04	2.1E-04	6.2E-04	4.4E-04	5.8E-04	3.8E-04	3.0E-04	N.D.
Nucleic acid	dTTP	C00459	HMDB01342	480.981	12.05	3.0E-03	3.8E-03	3.7E-03	1.0E-03	1.2E-03	9.1E-04	1.9E-03	1.7E-03	2.1E-03	N.D.	N.D.	N.D.
Nucleic acid	CTP	C00063	HMDB00082	481.976	12.29	6.3E-03	6.1E-03	5.9E-03	5.2E-03	6.1E-03	5.9E-03	4.8E-03	4.1E-03	4.4E-03	1.8E-03	1.4E-03	4.4E-04
Nucleic acid	UTP	C00075	HMDB00285	482.960	12.51	3.5E-02	3.7E-02	3.2E-02	2.9E-02	2.9E-02	3.0E-02	7.6E-02	6.6E-02	7.5E-02	1.1E-03	9.9E-04	3.1E-04
Nucleic acid	dATP	C00131	HMDB01532	489.992	11.65	9.4E-04	9.5E-04	7.8E-04	5.0E-04	4.9E-04	5.2E-04	1.2E-03	1.0E-03	1.0E-03	1.7E-04	N.D.	N.D.
Nucleic acid	ATP	C00002	HMDB00538	505.988	11.71	1.4E-01	1.3E-01	1.1E-01	1.2E-01	9.3E-02	1.0E-01	3.2E-01	2.5E-01	2.7E-01	3.6E-02	2.8E-02	6.0E-03
Nucleic acid	dGTP	C00286	HMDB01440	505.988	11.37	1.7E-03	1.3E-03	9.3E-04	4.1E-04	3.7E-04	2.4E-04	3.3E-04	N.D.	3.1E-04	N.D.	N.D.	N.D.
Nucleic acid	GTP	C00044	HMDB01273	521.984	11.38	1.1E-02	9.3E-03	8.3E-03	2.0E-02	1.5E-02	1.4E-02	7.7E-03	4.7E-03	5.5E-03	2.3E-03	1.7E-03	4.8E-04
Nucleic acid	Cytosine	C00380	HMDB00630	112.051	7.30	3.3E-04	3.4E-04	3.7E-04	3.4E-04	3.7E-04	2.7E-04	5.4E-04	4.6E-04	4.6E-04	3.7E-03	3.0E-03	2.4E-03
Nucleic acid	Adenine	C00147	HMDB00034	136.062	7.67	6.1E-03	6.0E-03	6.2E-03	3.8E-03	6.4E-03	5.4E-03	4.7E-03	5.1E-03	5.7E-03	3.1E-03	2.2E-03	2.4E-03
Nucleic acid	Guanine	C00242	HMDB00132	152.056	8.39	N.D.	N.D.	2.4E-04	9.1E-04	1.1E-03	6.8E-04	2.4E-03	2.2E-03	2.6E-03	1.2E-02	9.8E-03	9.3E-03
Nucleic acid	Thymidine	C00214	HMDB00273	243.098	23.04	1.7E-03	1.5E-03	1.7E-03	9.5E-04	1.0E-03	1.0E-03	1.6E-03	1.9E-03	1.7E-03	2.5E-03	1.9E-03	3.3E-03
Nucleic acid	Cytidine	C00475	HMDB00089	244.092	9.79	5.9E-04	7.2E-04	1.4E-03	3.6E-04	6.0E-04	6.2E-04	6.9E-04	6.0E-04	2.6E-04	6.5E-03	5.7E-03	3.4E-03
Nucleic acid	Uridine	C00299	HMDB00296	245.080	23.06	7.8E-04	7.3E-04	1.1E-03	8.8E-04	1.1E-03	1.0E-03	1.2E-03	1.3E-03	1.3E-03	2.9E-03	2.6E-03	1.9E-03
Nucleic acid	Adenosine	C00212	HMDB00050	268.104	9.99	1.1E-03	6.3E-04	1.2E-03	1.0E-03	5.5E-04	4.6E-04	1.1E-03	1.1E-03	1.2E-03	6.6E-03	7.7E-03	6.1E-03
Nucleic acid	Guanosine	C00387	HMDB00133	284.099	12.77	2.8E-04	3.1E-04	7.6E-04	2.8E-04	3.0E-04	3.8E-04	9.9E-04	6.5E-04	3.2E-04	2.0E-02	1.7E-02	9.0E-03
Others	Glycolic acid	C00160	HMDB00115	75.009	12.57	4.2E-03	N.D.	N.D.	N.D.	N.D.	N.D.	N.D.	N.D.	N.D.	N.D.	N.D.	N.D.
Others	Lactic acid	C00186,C00256,C01432	HMDB00190,HMDB01311	89.024	10.65	1.2E-02	5.8E-03	7.9E-03	8.4E-03	8.8E-03	1.4E-02	7.0E-03	6.8E-03	8.2E-03	5.7E-03	6.9E-03	4.9E-03
Others	Succinic semialdehyde	C00232	HMDB01259	101.024	9.84	2.0E-03	2.8E-03	2.5E-03	N.D.	N.D.	7.2E-04	N.D.	N.D.	N.D.	N.D.	N.D.	N.D.
Others	*N*-Formylglycine	No ID	No ID	102.019	10.69	1.1E-03	1.1E-03	1.9E-03	1.9E-04	3.6E-04	2.9E-04	9.2E-04	1.0E-03	8.1E-04	2.1E-04	2.2E-04	N.D.
Others	Glyceric acid	C00258	HMDB00139,HMDB06372	105.019	10.27	1.2E-03	1.7E-03	1.2E-03	5.0E-04	6.7E-04	6.5E-04	6.4E-04	6.2E-04	6.2E-04	N.D.	N.D.	4.5E-04
Others	Methyl sulfate	C02704	No ID	110.975	16.23	N.D.	1.4E-03	N.D.	1.1E-03	1.0E-03	N.D.	1.8E-03	1.4E-03	1.5E-03	N.D.	1.7E-03	1.3E-03
Others	XA0002**	No ID	No ID	110.985	15.78	1.5E-03	1.6E-03	N.D.	9.2E-04	1.0E-03	1.3E-03	1.7E-03	1.6E-03	1.8E-03	N.D.	N.D.	N.D.
Others	2-Furoic acid	C01546	HMDB00617	111.008	10.31	1.3E-03	1.2E-03	1.3E-03	7.7E-04	5.6E-04	6.5E-04	8.7E-04	7.5E-04	7.7E-04	8.6E-04	9.7E-04	9.3E-04
Others	4-Acetamidobutanoic acid	C02946	HMDB03681	144.065	8.40	6.2E-03	6.3E-03	5.6E-03	1.9E-03	2.2E-03	1.8E-03	8.7E-04	8.8E-04	7.2E-04	1.3E-04	1.4E-04	2.6E-04
Others	*N*-Formylaspartic acid	C01044	No ID	160.024	17.53	8.9E-03	8.3E-03	1.1E-02	1.0E-03	1.6E-03	1.7E-03	5.1E-04	N.D.	N.D.	N.D.	N.D.	N.D.
Others	*N*-Acetylasparagine	No ID	HMDB06028	173.056	8.42	3.7E-02	3.0E-02	4.3E-02	1.1E-03	1.8E-03	1.4E-03	1.1E-03	8.3E-04	1.0E-03	N.D.	N.D.	N.D.
Others	Formiminoglutamic acid	No ID	HMDB00854	173.056	8.13	3.2E-02	2.8E-02	3.8E-02	1.2E-03	1.8E-03	1.3E-03	1.0E-03	8.9E-04	9.0E-04	3.6E-04	2.0E-04	N.D.
Others	*N*-Acetylaspartic acid	C01042	HMDB00812	174.039	14.51	3.1E-03	6.1E-03	4.8E-03	8.0E-04	1.4E-03	1.4E-03	N.D.	N.D.	N.D.	N.D.	N.D.	N.D.
Others	*N*-Acetylglutamine	No ID	HMDB06029	187.072	8.02	1.1E-03	1.3E-03	1.5E-03	5.8E-04	4.4E-04	5.9E-04	3.3E-04	3.7E-04	3.6E-04	N.D.	N.D.	N.D.
Others	*N*-Acetylglutamic acid	C00624	HMDB01138	188.056	13.17	6.4E-03	6.8E-03	7.6E-03	6.6E-04	8.0E-04	8.8E-04	2.6E-04	N.D.	1.6E-04	N.D.	N.D.	N.D.
Others	Phenaceturic acid	C05598	HMDB00821	192.066	8.00	6.7E-04	5.9E-04	1.0E-03	6.7E-04	1.1E-03	1.1E-03	1.0E-03	8.4E-04	1.1E-03	N.D.	N.D.	N.D.
Others	Pantothenic acid	C00864	HMDB00210	218.102	7.54	1.3E-03	1.5E-03	1.5E-03	2.9E-03	3.2E-03	4.0E-03	2.3E-04	2.1E-04	2.5E-04	N.D.	N.D.	N.D.
Others	2-Deoxyglucose 6-phosphate	C06369	No ID	243.026	10.17	8.1E-04	8.3E-04	4.5E-04	2.8E-04	3.0E-04	2.2E-04	1.5E-04	N.D.	N.D.	N.D.	N.D.	3.8E-04
Others	Glucosamine 6-phosphate	C00352	HMDB01254	258.037	8.78	5.2E-03	6.2E-03	6.0E-03	1.4E-03	1.2E-03	1.3E-03	4.2E-04	4.8E-04	4.5E-04	N.D.	N.D.	N.D.
Others	Rhein	C10401	No ID	283.022	8.52	9.7E-04	1.1E-03	6.7E-04	4.2E-04	5.0E-04	5.4E-04	1.7E-04	1.7E-04	2.7E-04	N.D.	N.D.	N.D.
Others	*N*-Acetylmuramic acid	C02713	No ID	292.102	7.15	3.5E-04	3.4E-04	2.9E-04	1.6E-04	1.9E-04	1.7E-04	1.7E-04	1.8E-04	9.2E-05	1.3E-02	1.4E-02	1.5E-02
Others	*N*-Acetylglucosamine 6-phosphate	C00357	HMDB01062	300.046	9.04	1.8E-04	1.3E-04	1.3E-04	N.D.	N.D.	N.D.	N.D.	N.D.	N.D.	7.2E-04	8.7E-04	1.2E-03
Others	*N*-Acetylglucosamine 1-phosphate	C04256	HMDB01367	300.047	9.44	2.5E-03	2.0E-03	2.1E-03	4.6E-04	4.5E-04	4.4E-04	7.6E-05	9.8E-05	9.2E-05	2.4E-03	2.8E-03	3.3E-03
Others	XA0055**	No ID	No ID	368.998	14.32	1.6E-03	2.3E-03	3.1E-03	3.8E-04	3.8E-04	5.4E-04	N.D.	N.D.	N.D.	N.D.	N.D.	N.D.
Others	PRPP	C00119	HMDB00280	388.943	16.98	1.8E-03	2.3E-03	1.5E-03	2.8E-03	4.2E-03	4.9E-03	6.0E-04	6.6E-04	8.4E-04	N.D.	N.D.	N.D.
Others	FAD_divalent	C00016	HMDB01248	391.569	7.74	2.6E-03	2.8E-03	2.1E-03	2.1E-03	1.5E-03	1.6E-03	2.0E-03	1.8E-03	1.8E-03	1.5E-03	1.7E-03	1.2E-03
Others	Sucrose 6'-phosphate	C02591	No ID	421.074	8.44	2.1E-02	2.2E-02	2.3E-02	5.9E-03	5.5E-03	5.8E-03	2.8E-03	2.2E-03	2.8E-03	1.1E-04	1.7E-04	5.2E-04
Others	Trehalose 6-phosphate	C00689	HMDB01124	421.075	8.29	3.7E-03	4.2E-03	3.3E-03	N.D.	9.5E-05	1.6E-04	1.4E-04	2.1E-04	1.1E-03	N.D.	N.D.	N.D.
Others	Thiamine diphosphate	C00068	HMDB01372	423.028	7.84	1.9E-03	1.8E-03	1.6E-03	1.8E-03	1.6E-03	1.6E-03	2.1E-03	1.6E-03	2.5E-03	1.4E-03	1.6E-03	1.4E-03
Others	FMN	C00061	HMDB01520	455.095	8.22	1.1E-03	1.2E-03	1.1E-03	1.1E-03	9.0E-04	1.1E-03	8.4E-04	8.2E-04	8.0E-04	8.6E-04	9.2E-04	1.0E-03
Others	ADP-ribose	C00301	HMDB01178	558.063	8.36	8.2E-04	8.9E-04	6.5E-04	7.4E-04	5.6E-04	8.4E-04	4.8E-04	5.0E-04	2.8E-04	1.4E-04	1.6E-04	1.2E-04
Others	dTDP-glucose	C00842	HMDB01328	563.069	8.40	1.3E-02	1.3E-02	1.3E-02	1.9E-03	2.0E-03	1.9E-03	5.1E-04	6.5E-04	9.2E-04	N.D.	N.D.	N.D.
Others	UDP-glucose<br>UDP-galactose	C00029<br>C00052	HMDB00286<br>HMDB00302	565.048	8.52	7.5E-02	8.5E-02	7.8E-02	3.8E-02	3.7E-02	4.1E-02	1.5E-02	1.4E-02	2.0E-02	1.4E-04	1.1E-04	1.8E-04
Others	UDP-glucuronic acid	C00167	HMDB00935	579.028	10.93	3.6E-03	3.4E-03	2.9E-03	1.6E-03	1.5E-03	1.6E-03	1.2E-03	1.2E-03	1.5E-03	1.1E-04	N.D.	N.D.
Others	ADP-glucose<br>GDP-fucose	C00498<br>C00325	HMDB06557<br>HMDB01095	588.074	8.22	1.2E-03	1.0E-03	1.3E-03	3.5E-04	2.3E-04	2.5E-04	2.0E-04	1.9E-04	2.3E-04	N.D.	N.D.	N.D.
Others	GDP-glucose	C00394	HMDB03351	604.068	8.17	7.2E-04	1.1E-03	9.0E-04	9.2E-04	9.0E-04	8.2E-04	2.6E-04	2.3E-04	2.6E-04	N.D.	N.D.	N.D.

Others	UDP-*N*-acetylglucosamine	C00043	HMDB00290	606.075	8.32	1.6E-02	1.5E-02	1.5E-02	1.5E-02	1.5E-02	1.4E-02	2.9E-03	2.7E-03	3.0E-03	1.2E-03	1.4E-03	1.4E-03
Others	NAD$^+$	C00003	HMDB00902	662.103	6.45	5.0E-02	5.1E-02	5.1E-02	3.5E-02	3.3E-02	3.5E-02	2.1E-02	2.0E-02	2.3E-02	8.9E-03	1.0E-02	5.2E-03
Others	NADP$^+$	C00006	HMDB00217	742.069	9.26	7.6E-03	6.5E-03	5.0E-03	1.1E-02	7.5E-03	7.7E-03	5.5E-03	3.9E-03	5.2E-03	7.2E-04	9.0E-04	5.7E-04
Others	Urea	C00086	HMDB00294	61.039	22.00	1.3E-02	N.D.	1.7E-02	1.6E-02	N.D.	2.2E-02	2.4E-02	1.9E-02	N.D.	N.D.	1.7E-02	N.D.
Others	Ethanolamine	C00189	HMDB00149	62.061	6.36	N.D.	N.D.	N.D.	N.D.	2.5E-04	N.D.	N.D.	N.D.	N.D.	1.0E-03	1.2E-03	1.4E-03
Others	Isobutylamine	C02787	No ID	74.097	7.05	N.D.	N.D.	N.D.	N.D.	N.D.	2.1E-03	N.D.	N.D.	N.D.	N.D.	N.D.	N.D.
Others	Morpholine	C14452	HMDB31581	88.076	6.66	N.D.	N.D.	N.D.	N.D.	N.D.	N.D.	N.D.	N.D.	N.D.	1.9E-03	1.4E-03	1.6E-03
Others	Putrescine	C00134	HMDB01414	89.107	4.69	7.6E-03	8.6E-03	9.5E-03	3.4E-03	3.4E-03	4.0E-03	4.1E-03	3.8E-03	6.0E-03	4.3E-02	5.3E-02	3.5E-02
Others	β-Ala	C00099	HMDB00056	90.055	7.38	3.3E-04	3.7E-04	5.1E-04	5.5E-04	7.8E-04	1.1E-03	N.D.	N.D.	N.D.	8.1E-04	7.1E-04	2.8E-04
Others	3-Aminopropane-1,2-diol	C06057	No ID	92.071	7.40	N.D.	1.4E-04	N.D.	1.4E-04	N.D.	2.0E-04	2.4E-04	2.9E-04	3.4E-04	4.2E-04	4.7E-04	6.8E-04
Others	Glycerol	C00116	HMDB00131	93.055	23.02	9.4E-01	7.1E-01	1.0E+00	1.2E+00	1.3E+00	1.1E+00	1.5E+00	1.5E+00	1.7E+00	2.1E+00	1.6E+00	1.3E+00
Others	1-Aminocyclopropane-1-carboxylic acid	C01234	No ID	102.055	7.10	1.9E-02	1.7E-02	1.4E-02	3.7E-03	2.3E-03	4.6E-03	9.4E-03	9.1E-03	1.9E-02	N.D.	N.D.	N.D.
Others	Cadaverine	C01672	HMDB02322	103.123	4.98	N.D.	N.D.	N.D.	N.D.	N.D.	N.D.	N.D.	N.D.	N.D.	1.3E-03	1.9E-03	1.2E-03
Others	2-Aminobutyric acid	C02261,C02356	HMDB00452	104.071	9.75	9.2E-04	1.9E-03	2.0E-03	N.D.	N.D.	N.D.	N.D.	N.D.	N.D.	N.D.	N.D.	N.D.
Others	GABA	C00334	HMDB00112	104.071	7.74	3.8E-04	4.1E-04	5.0E-04	N.D.	N.D.	N.D.	N.D.	N.D.	N.D.	N.D.	N.D.	N.D.
Others	*N*-Methylalanine	C02721	No ID	104.071	10.39	4.5E-04	N.D.	5.6E-04	N.D.	N.D.	N.D.	7.4E-04	7.0E-04	1.3E-03	N.D.	N.D.	N.D.
Others	Choline	C00114	HMDB00097	104.107	6.89	1.9E-03	2.2E-03	3.0E-03	5.0E-03	5.4E-03	6.2E-03	1.6E-02	1.5E-02	1.7E-02	5.5E-02	4.8E-02	3.4E-02
Others	Diethanolamine	C06772	HMDB04437	106.086	7.69	1.0E-03	2.2E-03	1.7E-03	1.2E-03	1.6E-03	1.3E-03	1.5E-03	1.1E-03	2.5E-03	2.8E-03	4.0E-03	1.4E-03
Others	3-Amino-2-piperidone	No ID	HMDB00323	115.086	7.65	N.D.	N.D.	N.D.	8.5E-04	9.4E-04	8.5E-04	1.3E-03	1.2E-03	1.2E-03	9.1E-04	6.6E-04	6.4E-04
Others	2-Methylserine	C02115	No ID	120.065	10.51	2.7E-03	3.6E-03	4.8E-03	1.3E-03	1.2E-03	1.7E-03	N.D.	N.D.	N.D.	N.D.	N.D.	N.D.
Others	Homoserine	C00263	HMDB00719	120.065	10.20	6.9E-02	6.4E-02	4.6E-02	2.2E-02	2.6E-02	3.2E-02	4.8E-02	4.2E-02	3.6E-02	N.D.	N.D.	N.D.
Others	Betaine aldehyde_+H_2O	C00576	HMDB01252	120.102	7.50	1.3E-04	N.D.	N.D.	N.D.	N.D.	N.D.	5.1E-04	5.8E-04	4.4E-04	2.7E-04	2.0E-04	2.4E-04
Others	2-Amino-2-(hydroxymethyl)-1,3-propanediol	C07182	No ID	122.081	8.38	3.4E-04	5.4E-04	4.5E-04	N.D.	N.D.	N.D.	N.D.	N.D.	N.D.	1.1E-03	5.8E-04	4.8E-04
Others	Nicotinamide	C00153	HMDB01406	123.055	7.45	1.4E-03	1.5E-03	1.2E-03	1.5E-03	1.4E-03	1.4E-03	1.5E-03	1.0E-03	9.3E-04	6.1E-04	5.4E-04	N.D.
Others	Imidazole-4-acetic acid	C02835	HMDB02024	127.050	8.09	N.D.	N.D.	N.D.	4.5E-04	N.D.	2.4E-04	2.6E-04	N.D.	N.D.	6.8E-04	4.3E-04	3.8E-04
Others	XC0016**	No ID	No ID	129.065	8.86	3.3E-04	2.6E-04	4.5E-04	4.6E-04	6.2E-04	7.1E-04	9.5E-04	7.4E-04	9.5E-04	N.D.	N.D.	N.D.
Others	Pipecolic acid	C00408	HMDB00070,HMDB00716,HMDB05960	130.086	10.40	1.2E-02	1.4E-02	2.2E-02	9.5E-03	1.1E-02	1.1E-02	2.5E-02	2.2E-02	3.3E-02	2.7E-03	2.2E-03	1.1E-03
Others	XC0017**	No ID	No ID	130.097	7.87	8.9E-04	1.2E-03	1.4E-03	1.1E-03	1.3E-03	1.3E-03	8.7E-04	8.5E-04	8.0E-04	1.2E-03	1.0E-03	5.7E-04
Others	*N*-Acetylputrescine	C02714	HMDB02064	131.118	8.52	4.5E-04	3.7E-04	5.0E-04	N.D.	N.D.	N.D.	N.D.	N.D.	N.D.	N.D.	N.D.	2.6E-04
Others	5-Amino-4-oxovaleric acid	C00430	HMDB01149	132.066	8.06	1.2E-03	1.7E-03	2.7E-03	9.1E-04	1.2E-03	9.1E-04	8.7E-04	8.3E-04	6.3E-04	N.D.	N.D.	N.D.
Others	Alloisoleucine	No ID	HMDB00557	132.102	10.59	4.8E-04	3.4E-04	N.D.	4.9E-04	5.1E-04	4.5E-04	N.D.	N.D.	N.D.	N.D.	N.D.	N.D.
Others	Ornithine	C00077,C00515,C01602	HMDB00214,HMDB03374	133.097	6.85	5.2E-03	6.0E-03	8.3E-03	5.7E-04	5.6E-04	7.4E-04	8.0E-04	5.0E-04	5.5E-04	1.4E-03	7.9E-04	5.0E-04
Others	Urocanic acid	C00785	HMDB00301	139.050	8.36	1.9E-04	1.7E-04	9.8E-04	6.9E-04	6.9E-04	1.5E-03	3.7E-04	3.5E-04	7.3E-04	5.5E-04	5.7E-04	2.9E-04
Others	1-Methyl-4-imidazoleacetic acid	C05828	HMDB02820	141.066	8.36	N.D.	N.D.	3.0E-04	N.D.	N.D.	3.8E-04	N.D.	N.D.	N.D.	N.D.	N.D.	N.D.
Others	*N*-Ethylmaleimide_+H_2O	C02441	No ID	144.065	23.19	1.1E-03	6.3E-04	6.5E-04	4.9E-04	5.8E-04	5.1E-04	N.D.	N.D.	N.D.	N.D.	N.D.	N.D.
Others	4-Guanidinobutyric acid	C01035	HMDB03464	146.092	8.36	4.0E-04	4.1E-04	5.4E-04	7.0E-04	4.4E-04	5.1E-04	4.6E-04	4.8E-04	4.3E-04	5.3E-03	3.5E-03	3.4E-03
Others	*O*-Acetylserine	C00979	HMDB03011	148.060	12.86	3.3E-03	5.7E-03	6.4E-03	2.6E-04	3.6E-04	4.3E-04	5.9E-04	N.D.	N.D.	N.D.	N.D.	N.D.
Others	*N*-Acetylserine	No ID	HMDB02931	148.060	24.38	5.3E-03	6.6E-03	6.7E-03	5.8E-03	7.0E-03	7.0E-03	5.4E-03	5.2E-03	4.9E-03	1.1E-03	N.D.	N.D.
Others	Triethanolamine	C06771	No ID	150.112	8.31	2.2E-04	1.6E-04	4.5E-04	5.4E-04	3.7E-04	6.0E-04	2.7E-04	2.6E-04	3.3E-04	4.7E-04	3.7E-04	4.4E-04
Others	Ala-Ala	C00993	HMDB03459	161.092	9.36	4.0E-02	4.9E-02	5.1E-02	1.4E-02	1.4E-02	1.1E-02	4.8E-03	3.7E-03	3.6E-03	1.1E-03	6.0E-04	N.D.
Others	N^6-Methyllysine	C02728	HMDB02038	161.128	7.13	4.5E-03	5.9E-03	6.9E-03	2.5E-04	2.5E-04	1.8E-04	2.5E-04	1.6E-04	N.D.	4.9E-02	3.9E-02	3.0E-02
Others	2-Aminoadipic acid	C00956	HMDB00510	162.075	11.04	3.6E-03	3.7E-03	4.0E-03	1.9E-03	1.7E-03	2.0E-03	8.9E-04	6.5E-04	5.8E-04	N.D.	N.D.	N.D.
Others	*N*-Methylglutamic acid	C01046	No ID	162.076	13.36	5.8E-04	8.3E-04	9.3E-04	N.D.	N.D.	N.D.	N.D.	N.D.	N.D.	N.D.	N.D.	N.D.
Others	*O*-Acetylhomoserine	C01077	No ID	162.077	11.17	3.7E-02	6.3E-02	8.6E-02	1.5E-03	2.3E-03	3.0E-03	2.8E-04	3.4E-04	N.D.	N.D.	N.D.	N.D.
Others	Carnitine	C00318,C00487,C15025	HMDB00062	162.111	8.70	3.7E-04	N.D.	4.1E-04	N.D.	N.D.	3.6E-04	5.3E-04	3.8E-04	N.D.	N.D.	N.D.	N.D.
Others	5-Hydroxylysine	C16741	HMDB00450	163.107	6.98	7.1E-04	1.2E-03	1.4E-03	3.7E-04	3.9E-04	3.7E-04	N.D.	N.D.	N.D.	2.5E-03	2.4E-03	1.7E-03
Others	2-Deoxystreptamine	C02627	No ID	163.109	8.63	3.0E-04	4.3E-04	2.7E-04	N.D.	N.D.	N.D.	N.D.	N.D.	N.D.	6.7E-04	6.3E-04	3.2E-04
Others	Pterin	C00715	HMDB00802	164.057	10.40	6.0E-03	5.0E-03	4.7E-03	6.2E-03	5.7E-03	5.4E-03	1.1E-02	1.2E-02	1.2E-02	1.5E-03	1.2E-03	9.3E-04
Others	6-Hydroxydopamine	No ID	HMDB01537	170.081	9.35	2.8E-04	1.6E-04	3.9E-04	2.9E-04	4.9E-04	5.0E-04	6.7E-04	8.2E-04	5.9E-04	1.6E-03	1.1E-03	3.3E-04
Others	Pyridoxine	C00314	HMDB00239	170.082	8.72	N.D.	1.9E-04	3.3E-04	1.7E-04	4.6E-04	3.2E-04	N.D.	2.8E-04	4.5E-04	3.4E-04	7.6E-04	4.4E-04
Others	Indole-3-acetaldoxim<br>Indole-3-acetamide	C02937<br>C02693	No ID<br>No ID	175.086	23.05	2.1E-03	2.0E-03	1.8E-03	1.5E-03	N.D.	1.5E-03	1.8E-03	N.D.	N.D.	N.D.	1.6E-03	N.D.
Others	N^5-Ethylglutamine	C01047	No ID	175.107	11.54	1.3E-03	1.9E-03	1.9E-03	7.1E-04	9.1E-04	8.7E-04	N.D.	N.D.	N.D.	2.1E-03	1.5E-03	8.2E-04
Others	*N*-Acetylornithine	C00437	HMDB03357	175.107	9.71	1.5E-03	1.2E-03	1.4E-03	N.D.	N.D.	2.3E-04	N.D.	N.D.	N.D.	6.1E-03	4.2E-03	1.3E-03
Others	Citrulline	C00327	HMDB00904	176.103	11.21	1.2E-03	1.8E-03	3.3E-03	N.D.	N.D.	N.D.	6.8E-04	7.3E-04	4.7E-04	2.3E-03	1.2E-03	3.4E-04

Others	Gluconolactone	C00198	HMDB00150	179.054	23.58	N.D.	N.D.	N.D.	N.D.	N.D.	N.D.	N.D.	N.D.	N.D.	2.1E-03	1.8E-03	1.0E-03
Others	Xanthopterin	No ID	No ID	180.051	13.49	4.5E-04	4.1E-04	6.7E-04	8.1E-04	8.9E-04	6.6E-04	1.3E-03	1.4E-03	1.6E-03	5.2E-04	3.1E-04	1.6E-04
Others	Glucosamine	C00329	HMDB01514	180.087	9.34	N.D.	N.D.	N.D.	N.D.	N.D.	N.D.	N.D.	N.D.	N.D.	6.1E-04	4.9E-04	6.1E-04
Others	*N*-Methylnorsalsolinol	No ID	HMDB01189	180.102	8.98	2.8E-04	2.9E-04	2.8E-04	4.7E-04	4.0E-04	3.4E-04	8.8E-04	5.5E-04	6.2E-04	1.5E-04	N.D.	N.D.
Others	*N*,*N*-Dimethylhistidine	C04259	No ID	184.108	8.38	6.3E-04	7.3E-04	9.0E-04	1.2E-03	1.3E-03	1.3E-03	1.1E-03	9.3E-04	9.4E-04	3.5E-04	2.0E-04	N.D.
Others	N^{8}-Acetylspermidine	C01029	HMDB02189	188.175	6.27	2.1E-04	1.8E-04	N.D.	1.3E-04	1.1E-04	7.4E-05	N.D.	N.D.	N.D.	8.4E-04	7.3E-04	4.4E-04
Others	*N*-Acetyllysine	C12989	HMDB00446	189.123	9.89	1.6E-03	1.5E-03	1.5E-03	5.5E-04	N.D.	3.1E-04	5.5E-04	7.2E-04	7.5E-04	7.7E-04	7.4E-04	4.6E-04
Others	Gly-Leu	No ID	No ID	189.123	9.76	2.5E-03	2.4E-03	2.8E-03	N.D.	N.D.	N.D.	N.D.	N.D.	N.D.	1.8E-02	1.0E-02	6.0E-03
Others	N^{6}-Acetyllysine	C02727	HMDB00206	189.123	11.61	N.D.	N.D.	N.D.	N.D.	N.D.	N.D.	N.D.	N.D.	N.D.	1.3E-03	9.8E-04	9.3E-04
Others	N^{6},N^{6},N^{6}-Trimethyllysine	C03793	HMDB01325	189.159	7.22	N.D.	N.D.	N.D.	N.D.	N.D.	N.D.	1.7E-03	1.9E-03	2.0E-03	3.0E-04	3.1E-04	2.3E-04
Others	Gly-Asp	No ID	No ID	191.066	10.00	4.7E-04	4.1E-04	5.0E-04	2.9E-04	3.3E-04	2.1E-04	N.D.	N.D.	N.D.	N.D.	N.D.	N.D.
Others	2,6-Diaminopimelic acid	C00666	HMDB01370	191.101	9.00	1.2E-03	1.7E-03	2.9E-03	4.2E-04	N.D.	N.D.	N.D.	N.D.	7.8E-05	N.D.	N.D.	N.D.
Others	Carboxymethyllysine	No ID	No ID	205.118	9.48	3.3E-04	3.4E-04	3.8E-04	5.0E-04	4.1E-04	4.5E-04	N.D.	N.D.	N.D.	8.2E-04	6.5E-04	3.9E-04
Others	*N*-Acetylglucosylamine	C01239	HMDB01104	221.114	10.02	8.6E-04	1.2E-03	1.2E-03	N.D.	N.D.	N.D.	N.D.	N.D.	N.D.	4.2E-03	3.1E-03	1.3E-03
Others	*N*-Acetylglucosamine	C00140	HMDB00215	222.097	23.06	9.7E-04	1.1E-03	1.5E-03	8.5E-04	1.2E-03	1.1E-03	N.D.	N.D.	N.D.	1.7E-02	1.5E-02	1.3E-02
Others	N^{2}-Succinylornithine	No ID	HMDB01199	233.112	10.37	2.3E-04	N.D.	2.9E-04	3.8E-04	6.0E-04	3.6E-04	3.7E-04	4.2E-04	3.3E-04	N.D.	N.D.	N.D.
Others	Pyridoxamine 5'-phosphate	C00647	HMDB01555	249.063	10.61	6.4E-03	6.5E-03	8.1E-03	5.7E-03	8.3E-03	6.9E-03	1.1E-02	1.0E-02	1.3E-02	2.3E-02	2.2E-02	1.7E-02
Others	2'-Deoxyadenosine<br>5'-Deoxyadenosine	C00559<br>C05198	HMDB00101<br>HMDB01983	252.108	9.79	1.1E-03	1.1E-03	1.1E-03	6.4E-04	1.1E-03	7.3E-04	2.4E-04	2.6E-04	3.0E-04	4.1E-04	3.8E-04	2.6E-04
Others	XC0089**	No ID	No ID	255.099	9.62	2.7E-04	2.4E-04	2.7E-04	1.9E-04	3.1E-04	1.8E-04	2.0E-04	2.2E-04	2.5E-04	8.2E-04	5.3E-04	5.0E-04
Others	Dyphylline	C07819	No ID	255.106	23.08	5.5E-04	6.7E-04	1.4E-03	N.D.	N.D.	6.7E-04	1.6E-03	1.9E-03	1.9E-03	N.D.	6.3E-04	2.3E-03
Others	Glycerophosphocholine	C00670	HMDB00086	258.110	22.55	1.0E-03	1.1E-03	1.7E-03	1.7E-03	1.8E-03	2.1E-03	1.9E-03	2.0E-03	1.9E-03	8.6E-04	9.4E-04	1.4E-03
Others	Thiamine	C00378	HMDB00235	265.112	6.61	8.2E-05	1.0E-04	1.2E-04	1.6E-04	2.3E-04	2.3E-04	2.1E-04	3.7E-04	9.9E-04	5.5E-03	5.4E-03	5.6E-03
Others	Ophthalmic acid	No ID	HMDB05765	290.135	13.47	1.3E-03	1.9E-03	1.6E-03	1.4E-03	1.6E-03	1.9E-03	N.D.	N.D.	N.D.	N.D.	N.D.	N.D.
Others	Argininosuccinic acid	C03406	HMDB00052	291.129	9.53	6.3E-04	1.2E-03	3.0E-03	N.D.	N.D.	7.7E-05	1.9E-04	1.9E-04	N.D.	1.9E-04	1.3E-04	1.6E-04
Others	Glutathione (GSSG)_divalent	C00127	HMDB03337	307.083	12.38	1.4E-02	1.4E-02	9.5E-03	3.2E-02	3.9E-02	3.0E-02	2.0E-02	2.0E-02	1.9E-02	1.1E-03	1.6E-03	8.3E-05
Others	NMN	C00455	HMDB00229	335.063	21.46	4.6E-04	3.4E-04	3.4E-04	4.4E-04	3.7E-04	3.8E-04	1.1E-04	9.8E-05	1.0E-04	3.4E-04	2.8E-04	2.1E-04
Others	Thiamine phosphate	C01081	HMDB02666	345.078	10.86	9.8E-05	1.1E-04	1.3E-04	6.1E-05	8.3E-05	6.5E-05	8.7E-05	7.2E-05	N.D.	6.7E-03	7.0E-03	6.1E-03
Others	Riboflavin	C00255	HMDB00244	377.146	22.92	9.3E-04	9.0E-04	1.1E-03	1.3E-03	1.2E-03	1.3E-03	6.0E-04	6.0E-04	5.8E-04	8.0E-04	7.5E-04	1.2E-03
Others	*S*-Adenosylmethionine	C00019	HMDB01185	399.144	7.15	1.5E-04	1.4E-03	1.4E-02	1.1E-03	1.8E-03	7.8E-04	1.2E-03	1.8E-03	2.8E-03	N.D.	N.D.	N.D.

*, central, central metabolism; amino acid, proteogenic amino acids; nucleic acid, nucleic acids metabolism

**, Metabolites are unknown but are frequently detected on measurement performance of Human Metabolome Technologies and those molecular formulas can be estimated. The estimated molecular fomula and detected mode of unknown metabolites; XA0002, CH5O4P, anion; XA0055, C7H16O13P2, anion; XC0016, C5H8N2O2, cation; XC0017, C5H11N3O, cation; XC0089, C11H24N2O8, cation

N.D., not detected.

MT, migration time of peak (minutes).

Table S2 List of metabolites changed markedly after 5 days starvation in dark

Metabolites	Relative peak area*				Ratio** d5-dark / d0
	d0-starved cells		d5-dark starved cells		
	Mean	S.D.	Mean	S.D.	
Increased (ratio > 1)					
N[6]-Methyllysine	2.3×10^{-4}	3.1×10^{-5}	4.0×10^{-2}	7.8×10^{-3}	173
Thiamine phosphate	7.0×10^{-5}	9.3×10^{-6}	6.6×10^{-3}	3.7×10^{-4}	95
N-Acetylmuramic acid	1.7×10^{-4}	1.4×10^{-5}	1.4×10^{-2}	8.5×10^{-4}	82
Lys	5.1×10^{-3}	6.3×10^{-4}	4.1×10^{-1}	1.7×10^{-1}	80
N-Acetylornithine	7.7×10^{-5}	1.1×10^{-4}	3.8×10^{-3}	2.0×10^{-3}	50
Guanosine	3.2×10^{-4}	4.1×10^{-5}	1.6×10^{-2}	4.8×10^{-3}	49
CMP	4.1×10^{-4}	7.6×10^{-5}	1.5×10^{-2}	8.1×10^{-3}	37
Thiamine	2.1×10^{-4}	3.4×10^{-5}	5.5×10^{-3}	8.0×10^{-5}	27
GMP	5.1×10^{-4}	1.1×10^{-4}	1.3×10^{-2}	3.3×10^{-3}	26
AMP	4.6×10^{-3}	8.2×10^{-4}	1.1×10^{-1}	4.0×10^{-2}	25
IMP	1.1×10^{-4}	4.0×10^{-5}	2.0×10^{-3}	2.0×10^{-4}	18
N-Acetylglucosamine	1.0×10^{-3}	1.3×10^{-4}	1.5×10^{-2}	1.6×10^{-3}	15
Ethanolamine	8.2×10^{-5}	1.2×10^{-4}	1.2×10^{-3}	1.4×10^{-4}	15
Putrescine	3.6×10^{-3}	2.7×10^{-4}	4.4×10^{-2}	7.0×10^{-3}	12
Guanine	8.9×10^{-4}	1.6×10^{-4}	1.0×10^{-2}	1.4×10^{-3}	12
UMP	6.4×10^{-4}	1.4×10^{-4}	6.5×10^{-3}	3.5×10^{-3}	10
Adenosine	6.7×10^{-4}	2.4×10^{-4}	6.8×10^{-3}	6.5×10^{-4}	10
Cytidine	5.3×10^{-4}	1.2×10^{-4}	5.2×10^{-3}	1.3×10^{-3}	9.9
Cytosine	3.3×10^{-4}	4.2×10^{-5}	3.0×10^{-3}	5.1×10^{-4}	9.2
Choline	5.5×10^{-3}	4.6×10^{-4}	4.6×10^{-2}	8.6×10^{-3}	8.2
Decreased (ratio < 1)					
UDP-glucose UDP-galactose	3.9×10^{-2}	2.0×10^{-3}	1.4×10^{-4}	3.2×10^{-5}	0.0037
UDP-glucuronic acid	1.6×10^{-3}	4.6×10^{-5}	3.8×10^{-5}	5.3×10^{-5}	0.024
UTP	2.9×10^{-2}	8.2×10^{-4}	7.9×10^{-4}	3.4×10^{-4}	0.027
Glutathione (GSSG)_divalent	3.4×10^{-2}	3.7×10^{-3}	9.2×10^{-4}	6.2×10^{-4}	0.028
Ala-Ala	1.3×10^{-2}	1.6×10^{-3}	5.5×10^{-4}	4.3×10^{-4}	0.043

Sucrose 6'-phosphate	5.7×10^{-3}	1.6×10^{-4}	2.7×10^{-4}	1.8×10^{-4}	0.047
N-Acetylserine	6.6×10^{-3}	5.6×10^{-4}	3.8×10^{-4}	5.4×10^{-4}	0.058
Glu	1.7×10^{-1}	2.3×10^{-2}	1.1×10^{-2}	5.6×10^{-3}	0.064
NADP+	8.8×10^{-3}	1.7×10^{-3}	7.3×10^{-4}	1.4×10^{-4}	0.083
4-Acetamidobutanoic acid	1.9×10^{-3}	1.6×10^{-4}	1.8×10^{-4}	5.8×10^{-5}	0.090
UDP-N-acetylglucosamine	1.4×10^{-2}	2.4×10^{-4}	1.3×10^{-3}	9.8×10^{-5}	0.091
GTP	1.6×10^{-2}	2.8×10^{-3}	1.5×10^{-3}	7.5×10^{-4}	0.091
Pro	1.5×10^{-1}	2.1×10^{-2}	1.5×10^{-2}	3.9×10^{-3}	0.099
Thr	1.0×10^{-1}	1.0×10^{-2}	1.0×10^{-2}	2.6×10^{-3}	0.10
dATP	5.0×10^{-4}	1.4×10^{-5}	5.7×10^{-5}	8.1×10^{-5}	0.11
N-Methylnorsalsolinol	4.0×10^{-4}	5.1×10^{-5}	5.2×10^{-5}	7.3×10^{-5}	0.13
Formiminoglutamic acid	1.4×10^{-3}	2.4×10^{-4}	1.9×10^{-4}	1.5×10^{-4}	0.13
Phe	7.9×10^{-3}	1.3×10^{-3}	1.1×10^{-3}	1.2×10^{-4}	0.14
N,N-Dimethylhistidine	1.2×10^{-3}	6.0×10^{-5}	1.8×10^{-4}	1.4×10^{-4}	0.15
Tyr	4.4×10^{-3}	6.0×10^{-4}	6.6×10^{-4}	5.3×10^{-5}	0.15

*, Peak areas of CE-TOFMS were normalized with optical densities of the cultures.

**, Ratio of the relative peak area in the d5-dark starved cells / that in the d0-starved cells

Top 20 highest increased and decreased metabolites are listed. Data are presented as the means and standard deviations (S.D.) of three independent cultures.

Table S3 List of metabolites changed markedly after 5 days starvation in the light

Metabolites	Relative peak area*				Ratio** d5-light / d0
	d0-starved cells		d5-light starved cells		
	Mean	S.D.	Mean	S.D.	
Increased (ratio > 1)					
Dyphylline	2.2×10^{-4}	3.2×10^{-4}	1.8×10^{-3}	1.4×10^{-4}	8.0
Trehalose 6-phosphate	8.6×10^{-5}	6.7×10^{-5}	5.0×10^{-4}	4.5×10^{-4}	5.7
Argininosuccinic acid	2.6×10^{-5}	3.6×10^{-5}	1.3×10^{-4}	8.9×10^{-5}	4.9
1-Aminocyclopropane-1-carboxylic acid	3.5×10^{-3}	9.6×10^{-4}	1.3×10^{-2}	4.7×10^{-3}	3.6
Lys	5.1×10^{-3}	6.3×10^{-4}	1.7×10^{-2}	1.2×10^{-3}	3.4
N-Formylglycine	2.8×10^{-4}	7.0×10^{-5}	9.1×10^{-4}	8.0×10^{-5}	3.3
Choline	5.5×10^{-3}	4.6×10^{-4}	1.6×10^{-2}	8.7×10^{-4}	2.9
Gly	4.7×10^{-3}	6.5×10^{-4}	1.3×10^{-2}	1.1×10^{-3}	2.8
ATP	1.0×10^{-1}	1.0×10^{-2}	2.8×10^{-1}	3.0×10^{-2}	2.7
dCTP	2.0×10^{-4}	1.1×10^{-5}	5.5×10^{-4}	7.6×10^{-5}	2.7
Guanine	8.9×10^{-4}	1.6×10^{-4}	2.4×10^{-3}	1.8×10^{-4}	2.7
3-Aminopropane-1,2-diol	1.1×10^{-4}	8.4×10^{-5}	2.9×10^{-4}	4.1×10^{-5}	2.6
Pipecolic acid	1.0×10^{-2}	6.8×10^{-4}	2.7×10^{-2}	4.7×10^{-3}	2.6
Carnitine	1.2×10^{-4}	1.7×10^{-4}	3.0×10^{-4}	2.2×10^{-4}	2.6
Thiamine	2.1×10^{-4}	3.4×10^{-5}	5.3×10^{-4}	3.4×10^{-4}	2.5
UTP	2.9×10^{-2}	8.2×10^{-4}	7.3×10^{-2}	4.4×10^{-3}	2.5
Acetyl CoA_divalent	2.1×10^{-3}	3.4×10^{-4}	4.9×10^{-3}	1.1×10^{-3}	2.4
N-Acetyllysine	2.9×10^{-4}	2.2×10^{-4}	6.8×10^{-4}	8.8×10^{-5}	2.4
Methyl sulfate	7.0×10^{-4}	5.0×10^{-4}	1.6×10^{-3}	1.6×10^{-4}	2.2
dATP	5.0×10^{-4}	1.4×10^{-5}	1.1×10^{-3}	9.8×10^{-5}	2.2
Decreased (ratio < 1)					
Ribulose 5-phosphate	1.1×10^{-3}	7.2×10^{-5}	7.5×10^{-5}	1.1×10^{-4}	0.067
Pantothenic acid	3.4×10^{-3}	4.8×10^{-4}	2.3×10^{-4}	1.7×10^{-5}	0.067
Glu	1.7×10^{-1}	2.3×10^{-2}	1.4×10^{-2}	1.0×10^{-3}	0.081
O-Acetylhomoserine	2.2×10^{-3}	6.1×10^{-4}	2.1×10^{-4}	1.5×10^{-4}	0.094
Glucose 6-phosphate	1.8×10^{-2}	3.3×10^{-4}	1.9×10^{-3}	9.9×10^{-5}	0.11
Glucose 1-phosphate	9.9×10^{-4}	4.6×10^{-5}	1.1×10^{-4}	8.2×10^{-5}	0.12

N-Formylaspartic acid	1.5×10^{-3}	3.2×10^{-4}	1.7×10^{-4}	2.4×10^{-4}	0.12
Fructose 6-phosphate	4.0×10^{-3}	1.9×10^{-4}	4.8×10^{-4}	4.3×10^{-5}	0.12
Asp	9.1×10^{-3}	1.6×10^{-3}	1.4×10^{-3}	4.6×10^{-4}	0.16
Ala	7.2×10^{-3}	1.3×10^{-3}	1.2×10^{-3}	2.9×10^{-4}	0.17
PRPP	4.0×10^{-3}	8.5×10^{-4}	7.0×10^{-4}	1.0×10^{-4}	0.18
N-Acetylglutamic acid	7.8×10^{-4}	9.0×10^{-5}	1.4×10^{-4}	1.1×10^{-4}	0.18
2,6-Diaminopimelic acid	1.4×10^{-4}	2.0×10^{-4}	2.6×10^{-5}	3.7×10^{-5}	0.18
2-Deoxyglucose 6-phosphate	2.6×10^{-4}	3.4×10^{-5}	5.1×10^{-5}	7.2×10^{-5}	0.19
UDP-N-acetylglucosamine	1.4×10^{-2}	2.4×10^{-4}	2.9×10^{-3}	1.4×10^{-4}	0.20
N-Acetylglucosamine 1-phosphate	4.5×10^{-4}	6.0×10^{-6}	8.9×10^{-5}	9.4×10^{-6}	0.20
2-Phosphoglyceric acid	8.3×10^{-4}	6.1×10^{-5}	1.7×10^{-4}	1.2×10^{-4}	0.20
Sedoheptulose 7-phosphate	3.9×10^{-3}	1.1×10^{-4}	8.0×10^{-4}	1.3×10^{-4}	0.20
Ribose 5-phosphate	8.2×10^{-4}	1.4×10^{-5}	1.7×10^{-4}	1.2×10^{-4}	0.21
Asn	1.3×10^{-2}	1.6×10^{-3}	3.1×10^{-3}	4.0×10^{-4}	0.24

*, Peak areas of CE-TOFMS were normalized with optical densities of the cultures.

**, Ratio of the relative peak area in the d5-light starved cells / that in the d0-starved cells

Top 20 highest increased and decreased metabolites are listed. Data are presented as the means and standard deviations (S.D.) of three independent cultures.

Table S4 Concentrations of 60 metabolites quantified in this study. These data were used for Fig. 2 and Fig. S3.

Category*	Metabolite	pmol OD660-1											
		d0-starved cells			d5-light starved cells			d5-dark starved cells			growing cells		
		1	2	3	1	2	3	1	2	3	1	2	3
Central	Succinic acid	80	74	82	30	31	23	22	21	N.D.	640	544	630
Central	Malic acid	10	15	17	12	5.4	5.9	38	49	48	86	102	151
Central	Phosphoenolpyruvic acid	88	92	95	29	20	22	30	33	107	298	279	291
Central	Dihydroxyacetone phosphate	42	35	44	12	14	8.9	N.D.	N.D.	N.D.	127	153	182
Central	Glyceraldehyde 3-phosphate	22	23	17	N.D.	N.D.	N.D.	N.D.	N.D.	N.D.	56	53	41
Central	Glycerol 3-phosphate	183	158	76	104	146	154	68	101	214	148	138	84
Central	3-Phosphoglyceric acid	181	191	201	52	34	56	65	65	256	808	822	897
Central	2-Phosphoglyceric acid	27	25	23	N.D.	6.6	8.6	8.6	8.7	32	85	92	101
Central	Citric acid	32	21	26	17	12	13	23	23	13	141	141	117
Central	Ribose 5-phosphate	19	18	18	N.D.	6.4	5.4	8.2	7.5	9.4	99	98	103
Central	Ribulose 5-phosphate	26	30	30	5.8	N.D.	N.D.	22	27	36	1,255	1,421	1,671
Central	Fructose 6-phosphate	94	93	84	11	11	9.4	9.8	9.1	40	963	971	912
Central	Glucose 6-phosphate	439	432	420	43	45	48	58	46	224	4,099	4,328	4,230
Central	Glucose 1-phosphate	25	22	22	N.D.	3.7	4.3	N.D.	N.D.	11	183	198	185
Central	Sedoheptulose 7-phosphate	87	82	83	20	13	19	61	68	158	3,267	2,952	2,797
Central	Fructose 1,6-diphosphate	37	34	36	N.D.	N.D.	N.D.	N.D.	N.D.	29	95	150	231
Central	Acetyl CoA_divalent	38	25	33	103	64	65	16	13	1.9	100	98	96
Amino acid	Gly	39	55	50	121	130	148	117	101	60	169	218	272
Amino acid	Ala	50	35	34	8.9	5.6	5.5	54	50	31	337	521	609
Amino acid	Ser	95	115	111	52	45	47	21	21	18	490	474	575
Amino acid	Pro	489	619	450	358	334	438	67	54	34	361	402	472
Amino acid	Val	20	23	20	11	10	7.1	9.2	9.9	20	165	242	244
Amino acid	Thr	501	623	514	260	212	230	73	59	39	429	407	466
Amino acid	Ile	17	26	27	12	11	9.8	4.6	4.0	7.1	37	60	69
Amino acid	Leu	17	22	19	13	10	9.3	10	12	14	106	161	145
Amino acid	Asn	85	112	90	26	24	19	N.D.	N.D.	N.D.	133	172	170
Amino acid	Asp	45	65	69	13	7.7	6.6	20	18	11	805	717	1,132
Amino acid	Gln	62	85	71	48	40	34	N.D.	N.D.	N.D.	2,483	2,812	3,762
Amino acid	Lys	27	31	37	104	99	117	3,568	2,870	1,116	357	509	579
Amino acid	Glu	867	1,064	1,212	93	78	82	98	84	18	5,702	8,731	10,943

Amino acid	His	110	134	124	80	84	83	43	34	13	45	43	45
Amino acid	Phe	16	22	24	23	21	19	2.6	2.9	3.4	13	13	13
Amino acid	Arg	122	135	107	77	70	82	287	262	162	114	133	187
Amino acid	Tyr	16	22	22	7.9	7.9	7.6	3.3	2.9	2.7	19	20	26
Amino acid	Trp	1.6	2.0	1.9	2.2	1.9	2.6	1.8	1.4	1.2	1.2	1.6	1.7
Nucleic acid	CMP	9.5	11	15	15	12	11	202	352	760	21	20	21
Nucleic acid	UMP	12	10	17	20	15	22	62	104	231	10	15	18
Nucleic acid	AMP	77	62	96	100	86	114	1,148	1,831	2,782	64	65	84
Nucleic acid	IMP	3.0	5.7	2.7	N.D.	N.D.	N.D.	73	72	58	12	9.6	10
Nucleic acid	GMP	12	11	17	7.3	6.7	8.3	240	343	453	7.8	7.9	12
Nucleic acid	CDP	31	43	45	18	17	22	76	102	80	43	37	39
Nucleic acid	UDP	118	121	152	201	178	232	43	56	46	153	147	133
Nucleic acid	ADP	308	259	329	554	424	585	890	1,183	673	402	350	297
Nucleic acid	GDP	81	60	78	22	15	18	67	94	52	47	42	39
Nucleic acid	CTP	149	174	169	137	117	126	51	42	13	181	176	169
Nucleic acid	UTP	520	515	549	1,380	1,201	1,360	19	18	5.6	629	661	577
Nucleic acid	ATP	1,931	1,530	1,668	5,342	4,154	4,537	596	467	98	2,317	2,101	1,887
Nucleic acid	GTP	593	434	405	223	136	159	66	50	14	325	269	240
Nucleic acid	Cytosine	2.1	2.2	1.6	3.3	2.7	2.8	22	18	14	2.0	2.0	2.2
Nucleic acid	Adenine	17	28	24	21	23	25	14	9.8	10	27	27	28
Nucleic acid	Guanine	5.2	6.2	3.9	14	12	15	71	56	53	N.D.	N.D.	1.4
Nucleic acid	Thymidine	13	14	14	23	27	23	35	26	46	24	21	23
Nucleic acid	Cytidine	1.7	2.9	3.0	3.3	2.9	1.3	31	27	16	2.8	3.5	6.9
Nucleic acid	Uridine	13	17	15	18	20	20	44	39	29	12	11	17
Nucleic acid	Adenosine	3.8	2.1	1.7	4.3	4.2	4.5	24	29	23	4.3	2.3	4.5
Nucleic acid	Guanosine	1.5	1.6	2.0	5.2	3.4	1.7	107	91	47	1.5	1.6	4.0
Nucleic acid	dTDP	6.7	5.6	5.7	6.0	6.2	7.7	4.2	4.5	3.5	22	20	21
Nucleic acid	dCTP	4.9	4.6	5.3	15	11	14	9.2	7.4	N.D.	12	15	18
Nucleic acid	dTTP	20	24	18	38	35	43	N.D.	N.D.	N.D.	61	77	75
Nucleic acid	dATP	10	10	11	26	22	21	3.6	N.D.	N.D.	20	20	16

*, central, central metabolism; amino acid, proteogenic amino acids; nucleic acid, nucleic acids metabolism

N.D., not detected.

The following compounds were not detected in any sample tested in this study: compounds related to central metabolism were glyoxylic acid, pyruvic acid, 2-oxoglutaric acid, cis-aconitic acid, erythrose 4-phosphate, fumaric acid and isocitric acid; compounds related to amino acid metabolism were Cys and Met; compounds related to nucleic acid metabolism were inosine, thymine and uracil.
